# Functional mechanism of hsa-miR-128-3p in epithelial-mesenchymal transition of pancreatic cancer cells via ZEB1 regulation

**DOI:** 10.7717/peerj.12802

**Published:** 2022-02-03

**Authors:** Tianying Zheng, Wenfei Han, Aijun Wang, Yonggang Wang

**Affiliations:** Medical Oncology, Qilu Hospital of Shandong University, Jinan, Shandong, China

**Keywords:** Pancreatic cancer, Hsa-miR-128-3p, ZEB1, Epithelial-mesenchymal transition, Invasion, Migration

## Abstract

Pancreatic cancer (PC) often correlates with high mortality due to late diagnosis, rapid metastasis, and resistance to chemotherapy. miR-128-3p has been validated as a tumor suppressor in PC. This study explored the functional mechanism of miR-128-3p in epithelial-mesenchymal transition (EMT) of PC cells. Four PC cancer cell lines with different degrees of malignancy and normal pancreatic cells were selected to detect expressions of hsa-miR-128-3p and ZEB1 by RT-qPCR and Western blot. miR-128-3p mimic or si-ZEB1 was delivered into PANC-1 cells and miR-128-3p inhibitor or oe-ZEB1 was delivered into AsPC-1 cells. Expressions of epithelial and mesenchymal markers were analyzed by Western blot and cell fluorescence staining. The binding relationship between miR-128-3p and ZEB1 was examined by bioinformatics analysis and dual-luciferase assay, and verified by RT-qPCR and Western blot. PC cell invasion and migration were assessed by Transwell assays. Generally, hsa-miR-128-3p was poorly-expressed in PC cells. However, it was relatively more expressed in AsPC-1 cells with epithelial phenotypes relative to PANC-1 cells with mesenchymal phenotype, whereas ZEB1 expression showed opposite tendencies. PANC-1 cells transfected with miR-128-3p mimic or si-ZEB1 showed upregulated E-cadherin and downregulated N-cadherin, and transformed from mesenchymal phenotypes to epithelial phenotypes, with decreased invasion and migration, while opposite results occurred in AsPC-1 cells transfected with miR-128-3p inhibitor or oe-ZEB1. miR-128-3p targeted ZEB1. oe-ZEB1 antagonized the inhibition of miR-128-3p mimic on PANC-1 cell EMT, invasion, and migration, while si-ZEB1 reversed the facilitation of miR-128-3p inhibitor in AsPC-1 cells. In conclusion, miR-128-3p inhibited PC cell EMT, invasion, and migration by targeting ZEB1.

## Introduction

Pancreatic cancer (PC) is a prevalent malignant tumor of the digestive system (Aroldi et al., 2016). Despite considerable improvements in medical conditions, the 5-year survival rate of PC is no more than 8% (Morrison, Byrne & Vonderheide, 2018). PC is often diagnosed at the late stage and chances for cure are rare partly due to rapid progression and poor prognosis (Ansari et al., 2016; Lin et al., 2015). The invasion and metastasis of cancer cells is the main contributor to mortality in PC (Zhou et al., 2017). Epithelial-mesenchymal transition (EMT) occurs during dissemination of PC cells, which in turn facilitates the invasiveness of PC cells (Wang, Huang & Sun, 2017). Therefore, it’s of significant importance to clarify the EMT regulatory mechanism in PC, thus contributing to the control of PC progression and improvement of the long-term survival rate of PC patients.

MicroRNAs (miRNAs) are a group of small endogenous short noncoding RNAs that manipulate gene expression at post-transcriptional level by binding to the 3′ untranslated region (UTR) of the target mRNA (Saliminejad et al., 2019). miRNAs are involved in a wide range of cellular processes including cell growth, proliferation, differentiation, metabolism, and apoptosis (Zhang, Jiang & Liu, 2017a). There are a variety of differentially expressed miRNAs in the tumor tissues of PC patients (Sohrabi et al., 2021). The dual regulatory effect of multiple miRNAs on PC has been extensively reported, such as the inhibitory effect of miR-203a-3p on the proliferation, EMT, and apoptosis of PC cells *via* SLUG (An & Zheng, 2020) and the promotional effect of miR-135-5p on the migration, invasion, and EMT of PC cells by targeting NR3C2 (Zhang et al., 2017b). Thus, we speculated that some other miRNAs might participate in EMT regulation in PC. Hsa-miR-128-3p, a suppressor for a variety of tumors including PC, has been reported to be weakly expressed in tumor tissues of PC patients (Kim et al., 2019; Wang et al., 2021). Therefore, the close relation between miR-128-3p and EMT regulation in PC is easily inferable. Moreover, miR-128-3p limits metastasis and EMT in esophageal squamous cell cancer (ESCC) by targeting zinc finger E-box binding homeobox factor 1 (ZEB1) (Zhao et al., 2018), which is known as the EMT activator and a key factor in promoting metastasis in PC (Krebs et al., 2017). We herein postulated that hsa-miR-128-3p inhibits EMT, invasion, and migration of PC cells by repressing ZEB1 expression. The present study was carried out based on the aforementioned hypothesis and aimed to explore the functional mechanism of hsa-miR-128-3p in EMT of PC cells, thus providing a novel theoretical reference for PC pathogenesis and therapeutic targets.

## Material and methods

### Ethics statement

The experiment was conducted with the approval of the Ethics Committee of Qilu Hospital of Shandong University.

### Cell culture

Normal human pancreatic duct epithelial cell line (HPDE6c7) and human PC cell lines (AsPC-1, BxPC-3, CFPAC-1, and PANC-1) with different degrees of malignancy were procured from the cell bank of Chinese Academy of Science (Shanghai, China). All cells were cultured in Dulbecco’s modified Eagle’s medium (DMEM) supplemented with 10% fetal bovine serum (FBS) and 1% penicillin-streptomycin (Procell, Wuhan, Hubei, China) at 37 °C with 5% CO_2_ in a humidified incubator (Thermo Forma 3111; Thermo Fisher Scientific, Waltham, MA, USA). The medium was refreshed every two days. The cells were detached with 0.25% trypsin and passaged every 3 days. After 3 passages, the morphology of cells at the logarithmic growth phase was observed under the light microscope (CX23; Olympus, Tokyo, Japan) before further experimentation.

### Cell grouping and transfection

PANC-1 and AsPC-1 cells at the logarithmic growth phase were seeded in 6-well plates. Once obtaining 60–70% confluence, the cells were co-transfected with miR-128-3p mimic or miR-128-3p inhibitor, oe-ZEB1 lentivirus, or si-ZEB1 using Lipofectamine 2000 (YT1317; Yita, Beijing, China). miR-128-3p mimic, miR-128-3p inhibitor, oe-ZEB1 lentivirus, si-ZEB1, and their controls were synthesized by Biomics Biotech (Nantong, Jiangsu, China) and used as per the provided instructions. PANC-1 cells were divided into the following groups according to different transfections: the blank group, the miR-mimic NC group, the miR-mimic group, the si-NC group, the si-ZEB1 group, the miR-mimic + oe-NC group, and the miR-mimic + oe-ZEB1 group. AsPC-1 cells were assigned into the following groups according to different transfections: the blank group, the miR-inhi NC group, the miR-inhi group, the oe-NC group, the oe-ZEB1 group, the miR-inhi + si-NC group, and the miR-inhi + si-ZEB1 group. The best transfection condition and concentrations of transfection reagents were confirmed in preparatory experiments to guarantee transfection efficiency. RT-qPCR, Western blot, and further experimentation was conducted after 48-h transfection.

### Transwell assay

Cells were harvested after 48-h transfection and made into cell suspension at the concentration of at 1 × 10^5^ cells/mL. Then, 200 µL cell suspension was slowly seeded in the apical chamber coated or not coated by Matrigel (60 mg/well, BD Bioscience, Franklin Lakes, NJ, USA) and 700 µL medium containing 10% FBS was supplemented to the basolateral chamber. Both chambers were placed in a 37 °C incubator with 5% CO_2_ and saturated humidity. The non-invading and non-migrating cells in the apical chamber were removed using cotton swabs. Following 48-h incubation, cells were fixed with 4% paraformaldehyde and stained for 10 min with 0.1% crystal violet. The number of cells passing through the membrane pores was counted. Three random visual fields of each well were selected for observation. The invasion and migration rates of PC cells in different groups were determined.

### Dual-luciferase assay

The binding sites of miR-128-3p and ZEB1 were predicted on bioinformatics online software TargetScan (http://www.targetscan.org). The Psicheck reporter gene detection plasmids (GenePharma, Shanghai, China) of ZEB1-WT (wild-type) and ZEB1-MUT (mutant) were constructed and co-delivered into PANC-1 cells with miR-128-3p mimic, and into AsPC-1 cells with miR-128-3p inhibitor in strict conformity with the instructions. Following 48-h transfection, relative fluorescence intensities of firefly luciferase and ranilla luciferase were detected.

### Reverse transcription-quantitative polymerase chain reaction (RT-qPCR)

Cells at the logarithmic growth phase were harvested and total RNA was extracted using RNAsimple Total RNA kits (R401-01; Vazyme, Nanjing, Jiangsu, China) and reverse-transcribed into cDNA using Taqman^®^ MicroRNA Reverse Transcription kit (Invitrogen, Carlsbad, CA, USA). PCR was performed on the ABI Prism 7300 system (ABI, Foster City, CA, USA) under the following conditions: pre-denaturation at 95 °C for 10 min and 40 cycles of 90 °C for 10 s, 60 °C for 20 s, and 72 °C for 30 s. U6 or *β*-actin served as internal controls. Relative gene expressions were calculated with the 2^−ΔΔ*CT*^ method. The amplified primer sequences were synthesized by Sangon Biotech (Shanghai, China) and illustrated in Table 1.

### Western blot

Cells at the logarithmic growth phase were collected and lysed with radio immunoprecipitation assay lysis buffer (SS0501; SORFA, Beijing, China) to extract the total protein. The protein concentration was quantified using BCA kits (PC0020-500, Acmec, Shanghai, China). The protein samples were denatured by boiling, separated using sodium dodecyl sulfate-polyacrylamide gel electrophoresis, electrophoretically transferred onto the polyvinylidene fluoride membranes, and blocked with 5% skim milk for 1 h. Next, the membranes were incubated with rabbit anti-human primary antibodies anti-E-cadherin (1:10000, ab40772; Abcam, Cambridge, MA, USA), anti-N-cadherin (1:1000, ab207608; Abcam), anti-ZEB1 (1:1000, ab203829; Abcam), and *β*-actin (1:1000, ab8227, Abcam) at 4 °C overnight. After washing, the membranes were probed with HRP-labeled goat anti-rabbit secondary antibody IgG H&L (1:300, ab7090; Abcam) for 1 h. The membranes were visualized with enhanced chemiluminescence (ECL) and photographed. The gray value of the protein bands was analyzed using the Image-Pro 6.0 software with *β*-actin as the internal control.

### Cell fluorescence staining

Cell climbing sheets were prepared, placed in the culture plate, washed thrice with PBS, fixed in 4% paraformaldehyde for 15 min, and cleared with 0.1% Triton X-100 for 10 min. After three PBS washes, the sheets were blocked with 10% goat serum for 30 min and incubated with primary antibodies E-cadherin (1:10000, ab40772), N-cadherin (1:200, ab18203) at 4 °C overnight. Following three PBS washes, the sheets were incubated with secondary antibody Alexa Fluor 488 (1:1000, ab150077) and counterstained with Alexa Fluor 594 (1:200, ab195889) at room temperature for 2 h under conditions devoid of light. After washing with PBS three times, DAPI was used for nuclear counterstaining. The sheets were sealed with an anti-fluorescence quenching sealing liquid (P0131-5mL; Beyotime, Shanghai, China) after three PBS washes. Images were captured under a fluorescence microscope (CKX53, Olympus).

**Table 1:** Primer sequences.

Gene	Forward 5′-3′	Reverse 5′-3′
*hsa-miR-128-3p*	GGTCACAGTGAACCGGTC	GTGCAGGGTCCGAGGT
*ZEB1*	CAGCTTGATACCTGTGAATGGG	CAGCTTGATACCTGTGAATGGG
*U6*	GCGCGTCGTGAAGCGTTC	GTGCAGGGTCCGAGGT
*β-actin*	CTCCATCCTGGCCTCGCTGT	GCTGTCACCTTCACCGTTCC

**Notes.**

miR, microRNA; ZEB1, zinc finger E-box binding homeobox factor 1

### Statistical analysis

Data were processed and plotted using SPSS 21.0 (IBM Corp., Armonk, NY, USA) and GraphPad Prism8.0 (GraphPad Software Inc., San Diego, CA, USA) software. Data were described as mean ± standard deviation. Comparisons among multi-groups were analyzed using one-way analysis of variance (ANOVA) following by Tukey’s multiple comparisons test. The *p* value was obtained using two-sided tests. Differences were regarded as statistical significance with *p* < 0.05, and ns meant no significant difference.

## Results

### hsa-miR-128-3p was weakly expressed while ZEB1 was highly expressed in different PC cells

**Figure 1:**
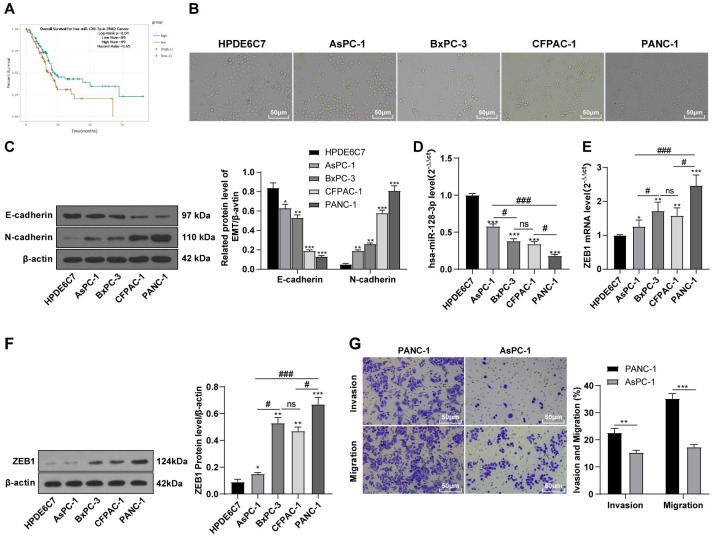
Hsa-miR-128-3p was weakly expressed while ZEB1 was highly expressed in different PC cells (A–F).

According to the ENCORI database (https://starbase.sysu.edu.cn/), low expression of miR-128-3p is an indicator of poor prognosis of PC patients (Fig. 1A). It’s been reported that EMT is a pivotal step in the invasion and metastasis of PC cells (Li et al., 2020; Rhim et al., 2012; Zhou et al., 2017). ZEB1 is a key transcriptional factor in EMT activation (Banerjee et al., 2021; Garinet et al., 2021). There is evidence that miR-128-3p inhibits metastasis and EMT in esophageal squamous-cell cancer *via* targeting ZEB1 (Zhao et al., 2018). Therefore, it is not unreasonable to assume a similar functional mechanism in PC. Four PC cell lines (PANC-1, CFPAC-1, BxPC-3, and AsPC-1) with different degrees of malignancy and normal PC cell line HPDE6c7 were selected to investigate the involvement of hsa-miR-128-3p and ZEB1 in EMT of PC cells. AsPC-1 cells and BxPC-3 cells were round- or ellipse-shaped with tight intercellular junctions and obvious cell polarity, while CFPAC-1 cells and PANC-1 cells showed a fusiform shape with loose intercellular junctions and loss of cell polarity (Fig. 1B). Western blot demonstrated increased E-cadherin (epithelial marker) and decreased N-cadherin (mesenchymal marker) in AsPC-1 and BxPC-3 cells, while the opposite was found in CFPAC-1 and PANC-1 cells (Fig. 1C, all *p* < 0.05). These results suggested that AsPC-1 cells and BxPC-3 cells were PC cell lines with epithelial phenotypes while CFPAC-1 cells and PANC-1 cells were with mesenchymal phenotypes.

RT-qPCR and Western blot showed decreased miR-128-3p expression (Fig. 1D), and increased ZEB1 expression in AsPC-1, BxPC-3, CFPAC-1 and PANC-1 cells relative to that in normal HPDE6c7 cells (Figs. 1E–1F, all *p* < 0.05). Among the PC cell lines, miR-128-3p expression was increased while ZEB1 expression was decreased in AsPC-1 cells with epithelial phenotypes relative to those in PANC-1 cells with mesenchymal phenotypes (all *p* < 0.05). Transwell assay showed stronger invasive and migrative abilities in PANC-1 cells relative to those in AsPC-1 cells (Fig. 1G, all *p* < 0.05). The above results demonstrated that hsa-miR-128-3p was poorly expressed while ZEB1 was highly expressed in PC cell lines and might be related to the EMT and malignant biological behaviors of PC cells.

### hsa-miR-128-3p overexpression partially inhibited the EMT, invasion, and migration of PC cells

**Figure 2:**
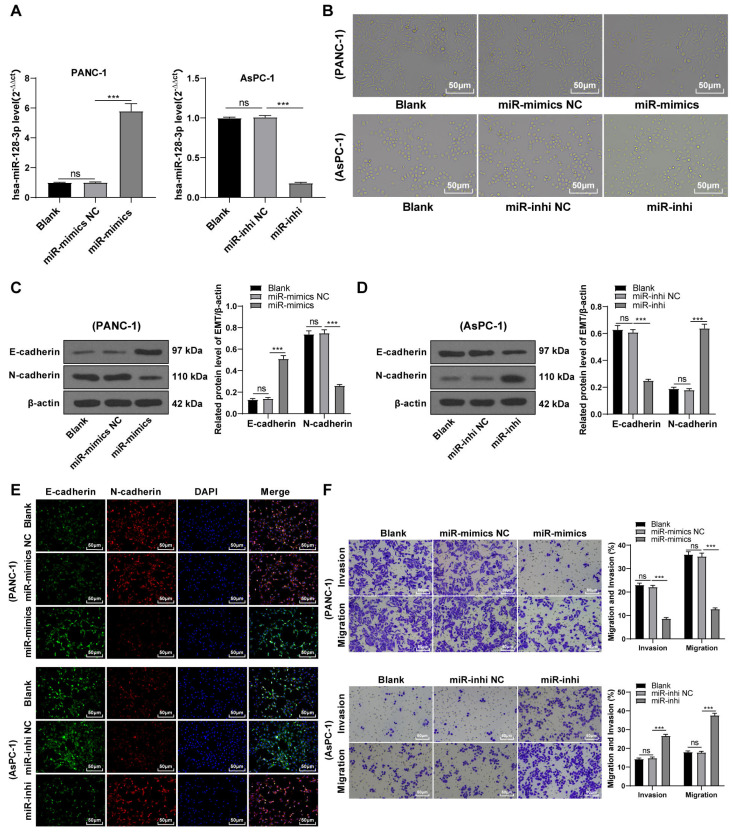
Hsa-miR-128-3p overexpression partially inhibited the EMT, invasion, and migration of PC cells (A–F).

To further validate the involvement of hsa-miR-128-3p in the EMT of PC cells, PANC-1 cells with relatively less expressed miR-128-3p and mesenchymal phenotypes and AsPC-1 cells with relatively more expressed miR-128-3p and epithelial phenotypes were selected for further experimentation. miR-128-3p mimic was delivered into PANC-1 cells to overexpress miR-128-3p and miR-128-3p inhibitor was delivered into AsPC-1 cells to downregulate miR-128-3p. The result showed upregulated expression of miR-128-3p in PANC-1 cells and downregulated expression of miR-128-3p in AsPC-1 cells after transfection (Fig. 2A, all *p* < 0.001), indicating successful transfection. PANC-1 cells transformed from mesenchymal phenotypes to epithelial phenotypes after miR-128-3p overexpression, while AsPC-1 cells transformed from epithelial phenotypes to mesenchymal phenotypes after miR-128-3p downregulation (Fig. 2B). Furthermore, Western blot showed that E-cadherin was increased and N-cadherin was decreased in PANC-1 cells after transfection with miR-128-3p mimic, while the opposite trend was found in AsPC-1 cells after transfection with miR-128-3p inhibitor (Figs. 2C–2D, all *p* < 0.001). The consistent tendency was found in cell fluorescence staining (Fig. 2E). Briefly, hsa-miR-128-3p overexpression inhibited the EMT of PC cells while downregulation of hsa-miR-128-3p facilitated the EMT of PC cells.

A close association between EMT and invasion and migration of PC cells has been documented (Sun, Chen & Dong, 2021; Xu et al., 2021). To further understand whether miR-128-3p affects invasion and migration of PC cells by manipulating the EMT process, we examined the effect of miR-128-3p on PC cell invasion and migration by Transwell assay, which showed decreased invasion and migration of PANC-1 cells transfected with miR-128-3p mimic, and increased invasion and migration of AsPC-1 cells transfected with miR-128-3p inhibitor (Fig. 2F, all *p* < 0.05). These results suggested that hsa-miR-128-3p overexpression repressed PC cell migration and invasion, whereas down-regulation of hsa-miR-128-3p enhanced PC cell migration and invasion.

### Silencing ZEB1 inhibited malignant biological behavior of PC cells

**Figure 3:**
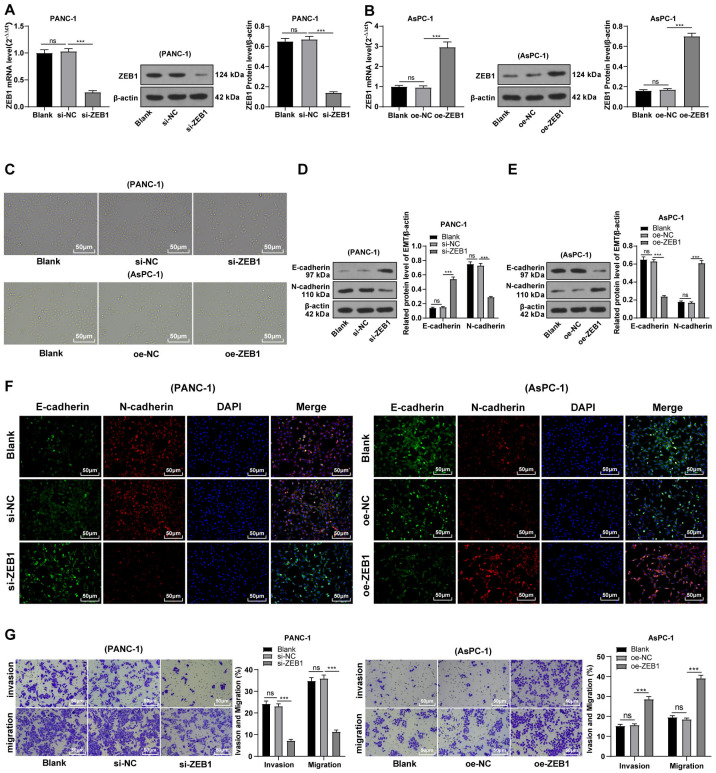
Silencing ZEB1 inhibited malignant biological behavior of PC cells (A–G). PANC-1 cell line was transfected with si-ZEB1, whereas AsPC-1 cell line was transfected with oe-ZEB1 for 48 h.

To explore the function of ZEB1 on EMT in PC, PANC-1 cells with mesenchymal phenotypes and higher ZEB1 expression were transfected with si-ZEB1, whereas AsPC-1 cells with epithelial phenotypes and lower ZEB1 expression were transfected with oe-ZEB1. RT-qPCR and Western blot were used to confirm the transfection efficiency (Figs. 3A–3B, all *p* < 0.05). Silencing ZEB1 promoted the transformation of PANC-1 cells from mesenchymal phenotype to epithelial phenotype, while ZEB1 overexpression facilitated the transformation of AsPC-1 cells from epithelial phenotype to mesenchymal phenotype (Fig. 3C). According to Western blot, E-cadherin was increased and N-cadherin was decreased in PANC-1 cells transfected with si-ZEB1, whereas E-cadherin was decreased and N-cadherin was increased in AsPC-1 cells transfected with oe-ZEB1 (Figs. 3D–3E, all *p* < 0.001). The same tendency was observed in cell fluorescence staining (Fig. 3F). These results together illustrated that silencing ZEB1 could suppress EMT of PC cells, while ZEB1 overexpression could facilitate EMT of PC cells.

To further investigate whether ZEB1 could affect PC cell invasion and migration *via* modulation of the EMT process, we detected the effect of ZEB1 on PC cell invasion and migration by Transwell assay, which exhibited decreased invasive and migrative abilities of PANC-1 cells transfected with si-ZEB1, and increased invasive and migrative abilities of AsPC-1 cells transfected with oe-ZEB1 (Fig. 3G, all *p* < 0.05). The above results indicated that silencing ZEB1 inhibited PC cell migration and invasion, whereas ZEB1 overexpression enhanced PC cell migration and invasion.

### hsa-miR-128-3p targeted ZEB1

**Figure 4:**
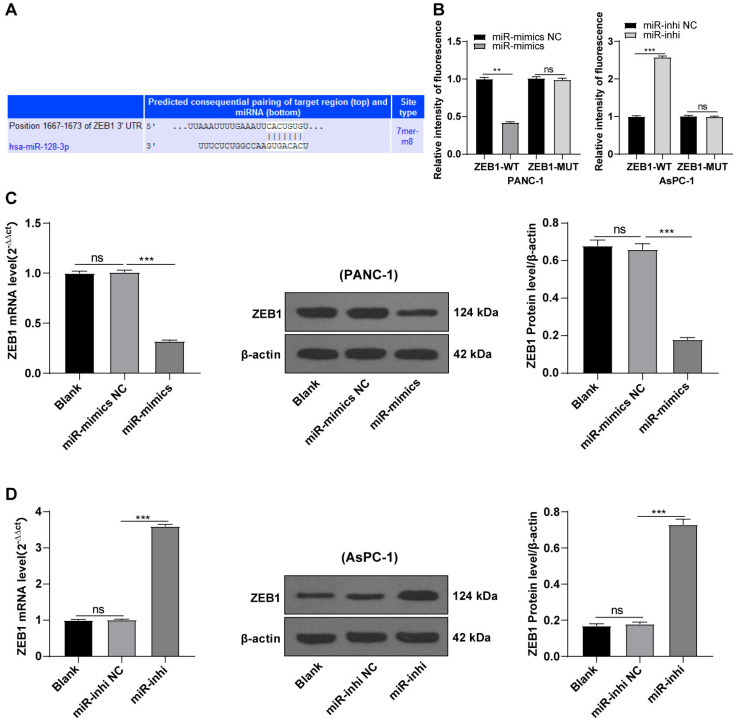
Hsa-miR-128-3p targeted ZEB1 (A–D).

Based on preliminary experimental results and background information, we speculated that miR-128-3p could target ZEB1. To testify our hypothesis, the binding sites of miR-128-3p and ZEB1 3′ UTR were predicted on the TargetScan database (http://www.targetscan.org), and the binding sequence was CACUGUG (Fig. 4A). The target relationship of miR-128-3p and ZEB1 was validated by a dual-luciferase assay, and miR-128-3p mimic in PANC-1 cells repressed the relative fluorescence intensity of ZEB1-WT reporter gene (Fig. 4B, *p* < 0.01), while miR-128-3p inhibitor in AsPC-1 cells enhanced the fluorescence intensity of ZEB1-WT (*p* < 0.001), but no apparent difference was observed in that of ZEB1-MUT. RT-qPCR and Western blot showed that ZEB1 was downregulated in PANC-1 cells after transfection with miR-128-3p mimic (Fig. 4C, all *p* < 0.001), and upregulated in AsPC-1 cells after transfection with miR-128-3p inhibitor (Fig. 4D, all *p* < 0.001). These results elicited that miR-128-3p targeted ZEB1.

### ZEB1 overexpression antagonized the inhibition of hsa-miR-128-3p overexpression on the EMT of PC cells

**Figure 5:**
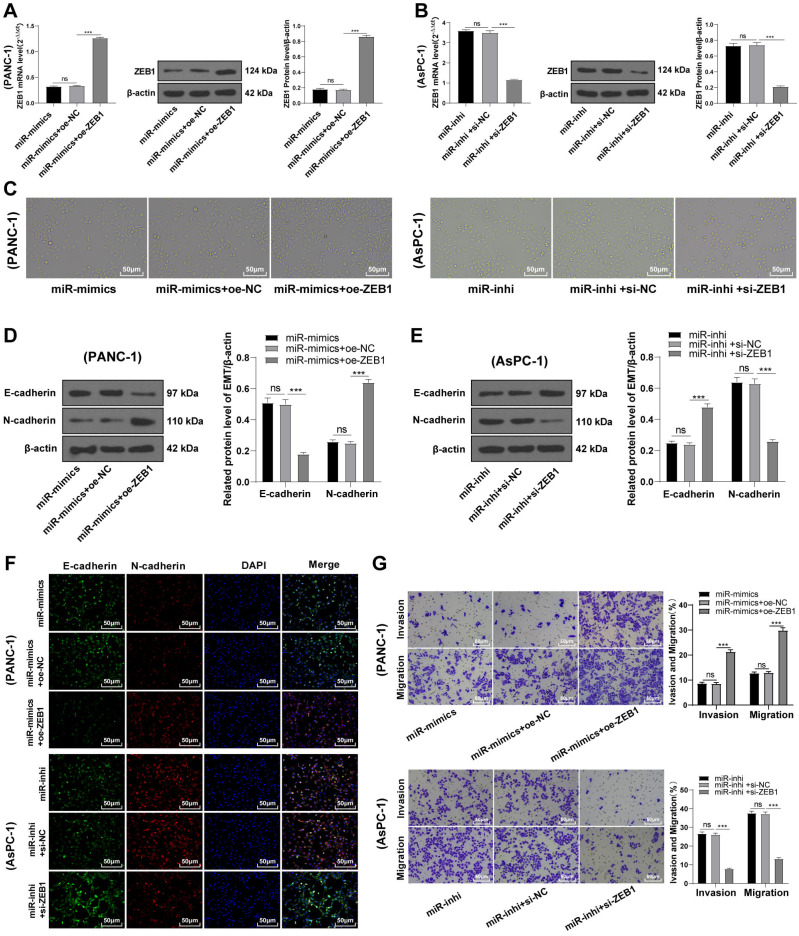
ZEB1 overexpression antagonized the inhibitory effect of hsa-miR-128-3p overexpression on epithelial mesenchymal transition of PC cells (A–G).

To identify the function of miR-128-3p on the EMT of PC cells *via* ZEB1, PANC-1 cells were transfected with oe-ZEB1 and miR-128-3p mimic, and AsPC-1 cells with si-ZEB1 and miR-128-3p inhibitor for 48 h. Western blot showed upregulated ZEB1 level in PANC-1 cells in the miR-mimic + oe-ZEB1 group (Fig. 5A, all *p* < 0.001), and downregulated ZEB1 level in AsPC-1 cells in the miR-inhi + si-ZEB1 group (Fig. 5B, all *p* < 0.001), indicating successful transfection of oe-ZEB1 or si-ZEB1. Furthermore, ZEB1 overexpression partially averted the inhibition of miR-128-3p overexpression on EMT and invasive and migrative abilities of PC cells. Conversely, silencing ZEB1 partially reversed the promotional effect of miR-128-3p downregulation on EMT and invasive and migrative abilities of PC cells (Figs. 5C–5G, all *p* < 0.001).

## Discussion

As a fatal malignancy, PC ranks the 3rd leading cause of cancer mortality and is projected to become the 2nd in the near future (Mizrahi et al., 2020). The potential role of miRNAs in the diagnosis, prognosis, and anti-cancer therapies of PC has been emphasized previously (Rawat et al., 2019). This study focused on investigating the function of miR-128-3p on EMT, invasion, and migration of PC cells by targeting ZEB1.

miR-128-3p has been indicated as a cancer suppressive factor and is capable of preventing EMT and cancer progression in diverse tumors (Cai et al., 2017; Frixa et al., 2018; Liu et al., 2019). During preliminary literature research and bioinformatics analysis, we identified the involvement of miR-128-3p in modulating PC development (Wang et al., 2021), suggesting the potential role of miR-128-3p as an inhibitory target in PC. Yet its functional mechanism and whether it poses an inhibitory impact on PC remains elusive. For this reason, we selected miR-128-3p as the target of this study. A correlation between weak expression of miR-128-3p and poor prognosis of PC has been indicated on the ENCORI database, which was a manifestation of miR-128-3p involvement in PC. Our results exhibited high miR-128-3p expression in normal pancreatic cells and low miR-128-3p expression in PC cell lines. Among the PC cell lines, miR-128-3p was more expressed in AsPC-1 cells relative to PANC-1 cells. EMT is a key mechanism in PC invasion and metastasis (Zhou et al., 2019). The switch from E-cadherin to N-cadherin is symbolic for EMT in cancer development (Zhou et al., 2017). Our results showed that E-cadherin was high and N-cadherin was weak in AsPC-1 cells and BxPC-3 cells, while the opposite was found in PANC-1 cells and CFPAC-1 cells, which further elicited epithelial phenotypes of AsPC-1 cells and BxPC-3 cells and mesenchymal phenotypes of PANC-1 cells and CFPAC-1 cells. Taken together, hsa-miR-128-3p was poorly expressed in PC cells and might be associated with EMT in PC.

A series of miRNAs have been reported to be involved in the invasion and migration of PC cells (Sohrabi et al., 2021). miR-128-3p overexpression can upregulate E-cadherin expression and inhibit EMT in colorectal cancer (Liu et al., 2019). To further validate the association between miR-128-3p and EMT in PC cells, miR-128-3p was overexpressed in PANC-1 cells and downregulated in AsPC-1 cells. Then, our result showed that PANC-1 cells transformed from mesenchymal phenotypes to epithelial phenotypes while AsPC-1 cells exhibited the opposite trend. Furthermore, E-cadherin was elevated and N-cadherin was reduced in PANC-1 cells after miR-128-3p overexpression, while AsPC-1 cells showed the opposite tendency after miR-128-3p downregulation. miR-128-3p upregulates expression of E-cadherin and prevents EMT formation in glioblastoma (Zhao et al., 2020). Similar to the previous findings, our results elucidated that overexpression of hsa-miR-128-3p inhibited EMT of PC cells, and downregulation of hsa-miR-128-3p facilitated it. EMT is closely associated with the invasion and migration of PC cells (Sun, Chen & Dong, 2021; Xu et al., 2021). We then investigated whether miR-128-3p affects the invasion and migration of PC cells *via* EMT. Our results revealed that the invasive and migrative abilities of PANC-1 cells were reduced after miR-128-3p overexpression while enhanced invasive and migrative abilities of AsPC-1 cells were observed after miR-128-3p downregulation. Upregulation of miR-128-3p could block the migration and invasion of cervical cancer cells (Wang et al., 2020). Consistently, our results elucidated that hsa-miR-128-3p overexpression repressed the invasion and migration of PC cells while downregulation of hsa-miR-128-3p promoted the invasion and migration of PC cells.

Subsequently, we explored the downstream mechanism of miR-128-3p in EMT of PC cells. Previous studies have indicated the roles of miR-128-3p downstream target genes, including ZEB1, CDC6, FOXO4, and SCAMP3 in EMT in several cancers (Bai et al., 2021; Kang, Zhang & Zhao, 2020). Among these target genes, ZEB1 has been reported to regulate the EMT of cancer cells in cervical carcinoma and esophageal squamous cell carcinoma (Feng et al., 2021; Zhao et al., 2018). Moreover, ZEB1 participates in the modulation of EMT in PC (Liu et al., 2021; Sheng et al., 2020a). However, whether miR-128-3p is associated with the action of ZEB1 on EMT in PC remains unknown. To verify this possibility, we selected ZEB1 as the downstream target gene. miR-128-3p was reported to inhibit metastasis and EMT in ESCC by targeting ZEB1 (Zhao et al., 2018). According to the ENCORI database, there was a weak negative correlation between ZEB1 and miR-128-3p. We herein predicted the binding sites of miR-128-3p and ZEB1 3′ UTR and verified their target relationship using dual-luciferase reporter assay. ZEB1 was downregulated in PANC-1 cells after miR-128-3p overexpression and upregulated in AsPC-1 cells after miR-128-3p downregulation. ZEB1 was accepted as the direct target of miR-128-3p (Zhang et al., 2020), which was further validated in our study.

ZEB1 could promote the E-cadherin expression and suppress the EMT of breast cancer cells (Wu et al., 2020). We observed increased expression of ZEB1 in PC cells, especially in PANC-1 cells with mesenchymal phenotypes, which was similar to the conclusion of a former study (Liu et al., 2020). To identify the function of ZEB1 in the EMT of PC cells, ZEB1 was overexpressed in PANC-1 cells and silenced in AsPC-1 cells. The results unraveled that ZEB1 overexpression promoted the transformation of PANC-1 cells from mesenchymal phenotypes to epithelial phenotypes, whereas silencing ZEB1 facilitates the transformation of AsPC-1 cells from epithelial phenotypes to mesenchymal phenotypes. Furthermore, silencing ZEB1 suppressed EMT, invasion, and migration of PC cells, whereas ZEB1 overexpression enhanced EMT, migration, and invasion of PC cells. Subsequently, we conducted joint treatment of oe-ZEB1 and miR-128-3p mimic in PANC-1 cells and joint treatment of si-ZEB1 and miR-128-3p inhibitor in AsPC-1 cells. As expected, ZEB1 overexpression annulled the inhibitory function of miR-128-3p overexpression on EMT, invasion, and migration of PC cells, while ZEB1 depletion averted the promotional function of miR-128-3p downregulation on EMT, invasion, and migration of PC cells. ZEB1 induces EMT and thus promotes PC cell migration and invasion (Sheng et al., 2020b). Based on the finding of a previous study, it’s further validated that ZEB1 overexpression abrogated the inhibition of miR-128-3p overexpression on EMT, invasion, and migration of PC cells.

To summarize, this study for the first time explored the functional mechanism of miR-128-3p in EMT, invasion, and migration of PC cells by targeting ZEB1 and thus provided a new theoretical basis for the mechanism of EMT of PC cells and insights into early diagnosis and therapeutic targets of PC. The current study has several limitations, though. First, the regulatory effect of miR-128-3p on PC cells was not investigated at the clinical level. Second, a limited number of PC cell lines was studied. Additionally, the animal experiment was not conducted to validate the regulation of miR-128-3p on PC cells *in vivo*. We will further investigate the expression levels of miR-128-3p and ZEB1 and their correlation in clinical samples in future studies. Apart from ZEB1, there are too many genes such as ACTR 3 and FGFR2c (Hu et al., 2021; Ranieri et al., 2021) that play regulatory roles in EMT in PC to list. These are not the downstream targets of miR-128-3p and are associated with other regulatory signaling pathways that fall outside of this study. Future investigations might study the function and mechanism of other target genes in PC. It would be of significance to investigate the interactions of multiple miRNAs, target genes, and pathways in PC based on clinical analysis in future studies.

##  Supplemental Information

10.7717/peerj.12802/supp-1 Supplemental Information 1 Correlation between miR-128-3p expression and poor prognosis of PC was analyzed in the ENCORI database

10.7717/peerj.12802/supp-2 Supplemental Information 2 The morphology of normal pancreatic cell line HPDE6C7 was observed under the light microscope

10.7717/peerj.12802/supp-3 Supplemental Information 3 The morphology of PC cell line AsPC-1 was observed under the light microscope

10.7717/peerj.12802/supp-4 Supplemental Information 4 The morphology of PC cell line BxPC-3 was observed under the light microscope

10.7717/peerj.12802/supp-5 Supplemental Information 5 The morphology of PC cell line CFPAC-1 was observed under the light microscope

10.7717/peerj.12802/supp-6 Supplemental Information 6 The morphology of PC cell line PANC-1 was observed under the light microscope

10.7717/peerj.12802/supp-7 Supplemental Information 7 Expressions of epithelial phenotype marker E-cadherin and mesenchymal phenotype marker N-cadherin were determined by Western blot

10.7717/peerj.12802/supp-8 Supplemental Information 8 The morphology of AsPC-1-Blank group cell was observed under the light microscope

10.7717/peerj.12802/supp-9 Supplemental Information 9 The morphology of AsPC-1-miR-inhi_NC group cell was observed under the light microscope

10.7717/peerj.12802/supp-10 Supplemental Information 10 The morphology of AsPC-1-miR-inhi group cell was observed under the light microscope

10.7717/peerj.12802/supp-11 Supplemental Information 11 The morphology of PANC-1-Blank group cell was observed under the light microscope

10.7717/peerj.12802/supp-12 Supplemental Information 12 The morphology of PANC-1-miR-mimics_NC group cell was observed under the light microscope

10.7717/peerj.12802/supp-13 Supplemental Information 13 The morphology of PANC-1-miR-mimics group cell was observed under the light microscope

10.7717/peerj.12802/supp-14 Supplemental Information 14 Expressions of E-cadherin and N-cadherin in PANC-1 cells were determined by Western blot

10.7717/peerj.12802/supp-15 Supplemental Information 15 Expressions of E-cadherin and N-cadherin in AsPC-1 cells were determined by Western blot

10.7717/peerj.12802/supp-16 Supplemental Information 16 Invasive ability of PANC-1-Blank group cell were evaluated by Transwell assays

10.7717/peerj.12802/supp-17 Supplemental Information 17 Invasive ability of PANC-1-miR-mimics_NC group cell were evaluated by Transwell assays

10.7717/peerj.12802/supp-18 Supplemental Information 18 Invasive ability of PANC-1-miR-mimics group cell were evaluated by Transwell assays

10.7717/peerj.12802/supp-19 Supplemental Information 19 Migrating ability of PANC-1-Blank group cell were evaluated by Transwell assays

10.7717/peerj.12802/supp-20 Supplemental Information 20 Migrating ability of PANC-1-miR-mimics_NC group cell were evaluated by Transwell assays

10.7717/peerj.12802/supp-21 Supplemental Information 21 Migrating ability of PANC-1-miR-mimics group cell were evaluated by Transwell assays

10.7717/peerj.12802/supp-22 Supplemental Information 22 Invasive ability of AsPC-1-Blank group cell were evaluated by Transwell assays

10.7717/peerj.12802/supp-23 Supplemental Information 23 Invasive ability of AsPC-1-miR-inhi_NC group cell were evaluated by Transwell assays

10.7717/peerj.12802/supp-24 Supplemental Information 24 Invasive ability of AsPC-1-miR-inhi group cell were evaluated by Transwell assays

10.7717/peerj.12802/supp-25 Supplemental Information 25 Migrating ability of AsPC-1-Blank group cell were evaluated by Transwell assays

10.7717/peerj.12802/supp-26 Supplemental Information 26 Migrating ability of AsPC-1-miR-inhi_NC group cell were evaluated by Transwell assays

10.7717/peerj.12802/supp-27 Supplemental Information 27 Migrating ability of AsPC-1-miR-inhi group cell were evaluated by Transwell assays

10.7717/peerj.12802/supp-28 Supplemental Information 28 Correlation between ZEB1 expression and poor prognosis of PC was analyzed in the ENCORI database

10.7717/peerj.12802/supp-29 Supplemental Information 29 Correlation between miR-128-33p and ZEB1 was analyzed in the ENCORI database

10.7717/peerj.12802/supp-30 Supplemental Information 30 Binding sites of miR-128-3p and ZEB1 3′UTR were predicted on the TargetScan database

10.7717/peerj.12802/supp-31 Supplemental Information 31 Protein expression of ZEB1 in PANC-1 cells was detected by Western blot

10.7717/peerj.12802/supp-32 Supplemental Information 32 Protein expression of ZEB1 in AsPC-1 cells was detected by Western blot

10.7717/peerj.12802/supp-33 Supplemental Information 33 ZEB1 expression in PANC-1 cells was detected by Western blot

10.7717/peerj.12802/supp-34 Supplemental Information 34 ZEB1 expression in AsPC-1 cells was detected by Western blot

10.7717/peerj.12802/supp-35 Supplemental Information 35 The morphology of AsPC-1-miR-inhi_+si-NC group cell was observed under the light microscope

10.7717/peerj.12802/supp-36 Supplemental Information 36 The morphology of AsPC-1-miR-inhi_+si-ZEB1 group cell was observed under the light microscope

10.7717/peerj.12802/supp-37 Supplemental Information 37 The morphology of AsPC-1-miR-inhi group cell was observed under the light microscope

10.7717/peerj.12802/supp-38 Supplemental Information 38 The morphology of PANC-1-miR-mimics group cell was observed under the light microscope

10.7717/peerj.12802/supp-39 Supplemental Information 39 The morphology of PANC-1-miR-mimics+oe-NC group cell was observed under the light microscope

10.7717/peerj.12802/supp-40 Supplemental Information 40 The morphology of PANC-1-miR-mimics+oe-ZEB1 group cell was observed under the light microscope

10.7717/peerj.12802/supp-41 Supplemental Information 41 Expressions of markers of epithelial mesenchymal transition in PANC-1 cells were determined by Western blot

10.7717/peerj.12802/supp-42 Supplemental Information 42 Expressions of markers of epithelial mesenchymal transition in AsPC-1 cells were determined by Western blot

10.7717/peerj.12802/supp-43 Supplemental Information 43 Invasive ability of AsPC-1-miR-inhi group cell were evaluated by Transwell assays

10.7717/peerj.12802/supp-44 Supplemental Information 44 Invasive ability of AsPC-1-miR-inhi+si-NC group cell were evaluated by Transwell assays

10.7717/peerj.12802/supp-45 Supplemental Information 45 Invasive ability of AsPC-1-miR-inhi+si-ZEB1 group cell were evaluated by Transwell assays

10.7717/peerj.12802/supp-46 Supplemental Information 46 Migrating ability of AsPC-1-miR-inhi group cell were evaluated by Transwell assays

10.7717/peerj.12802/supp-47 Supplemental Information 47 Migrating ability of AsPC-1-miR-inhi+si-NC group cell were evaluated by Transwell assays

10.7717/peerj.12802/supp-48 Supplemental Information 48 Migrating ability of AsPC-1-miR-inhi+si-ZEB1 group cell were evaluated by Transwell assays

10.7717/peerj.12802/supp-49 Supplemental Information 49 Original data

## Additional Information and Declarations

### Data Availability

The following information was supplied regarding data availability: The raw data are available in the Supplemental File.
