# Supplementary figures and images for: Functional mechanism of hsa-miR-128-3p in epithelial-mesenchymal transition of pancreatic cancer cells via ZEB1 regulation
Source: PeerJ. 2022 Feb 3;10:e12802. doi: 10.7717/peerj.12802 (PMC8818272; doi:10.7717/peerj.12802)

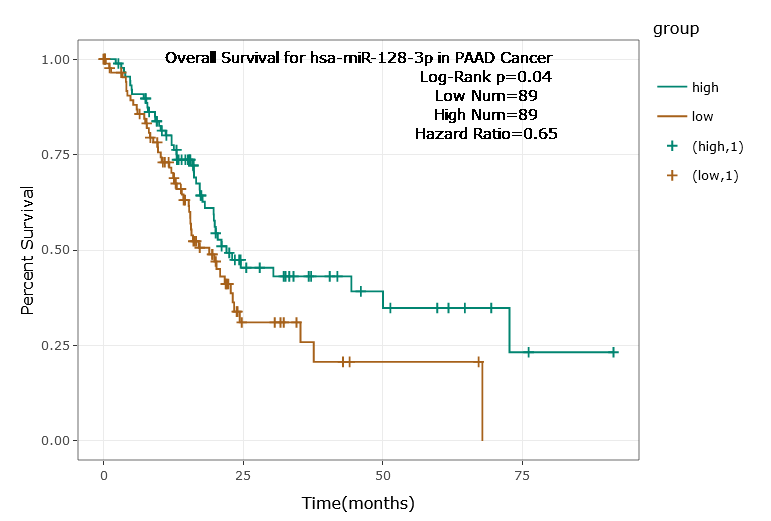

Supplement: Supplemental Information 1 [file peerj-10-12802-s001.png]

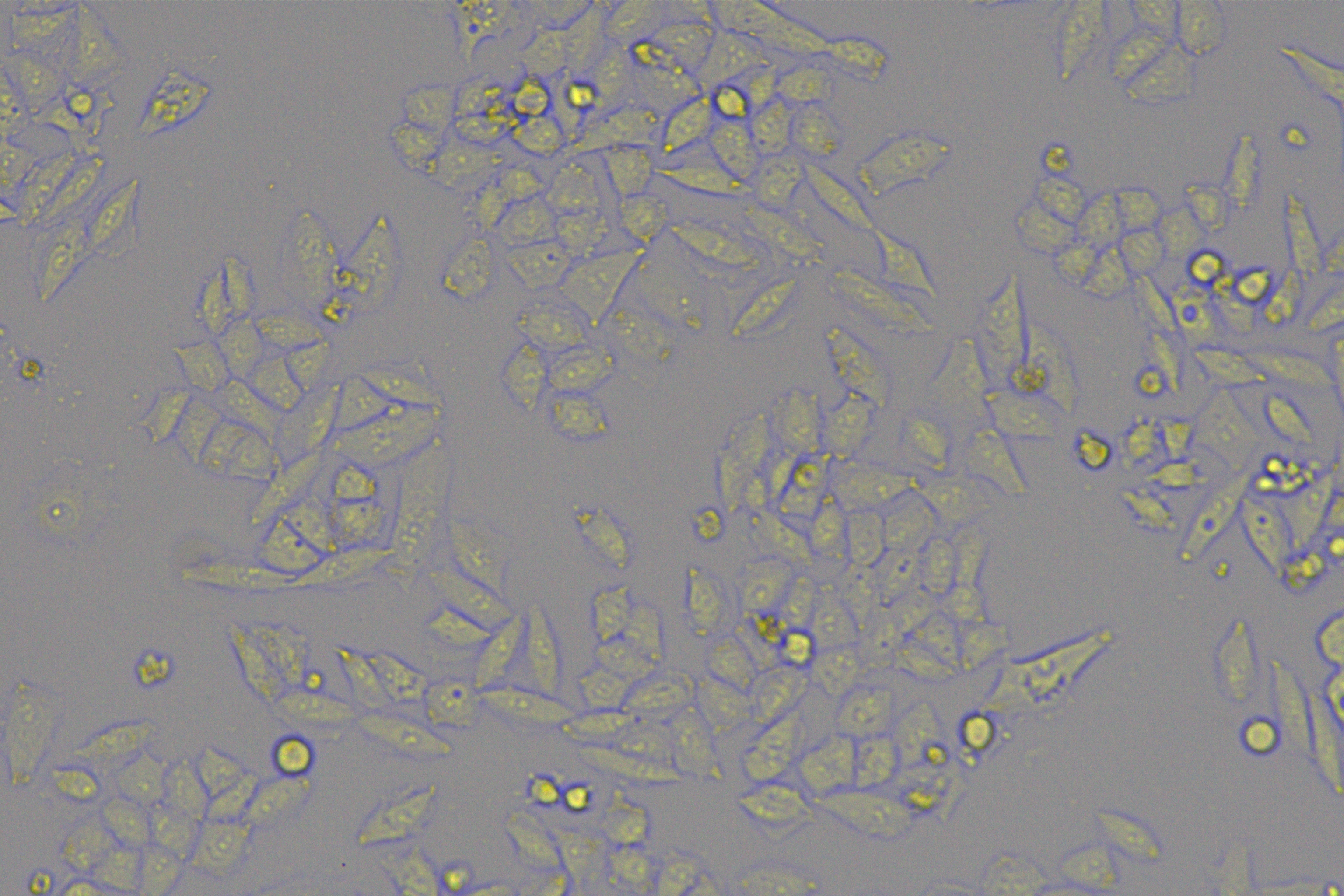

Supplement: Supplemental Information 2 [file peerj-10-12802-s002.jpg]

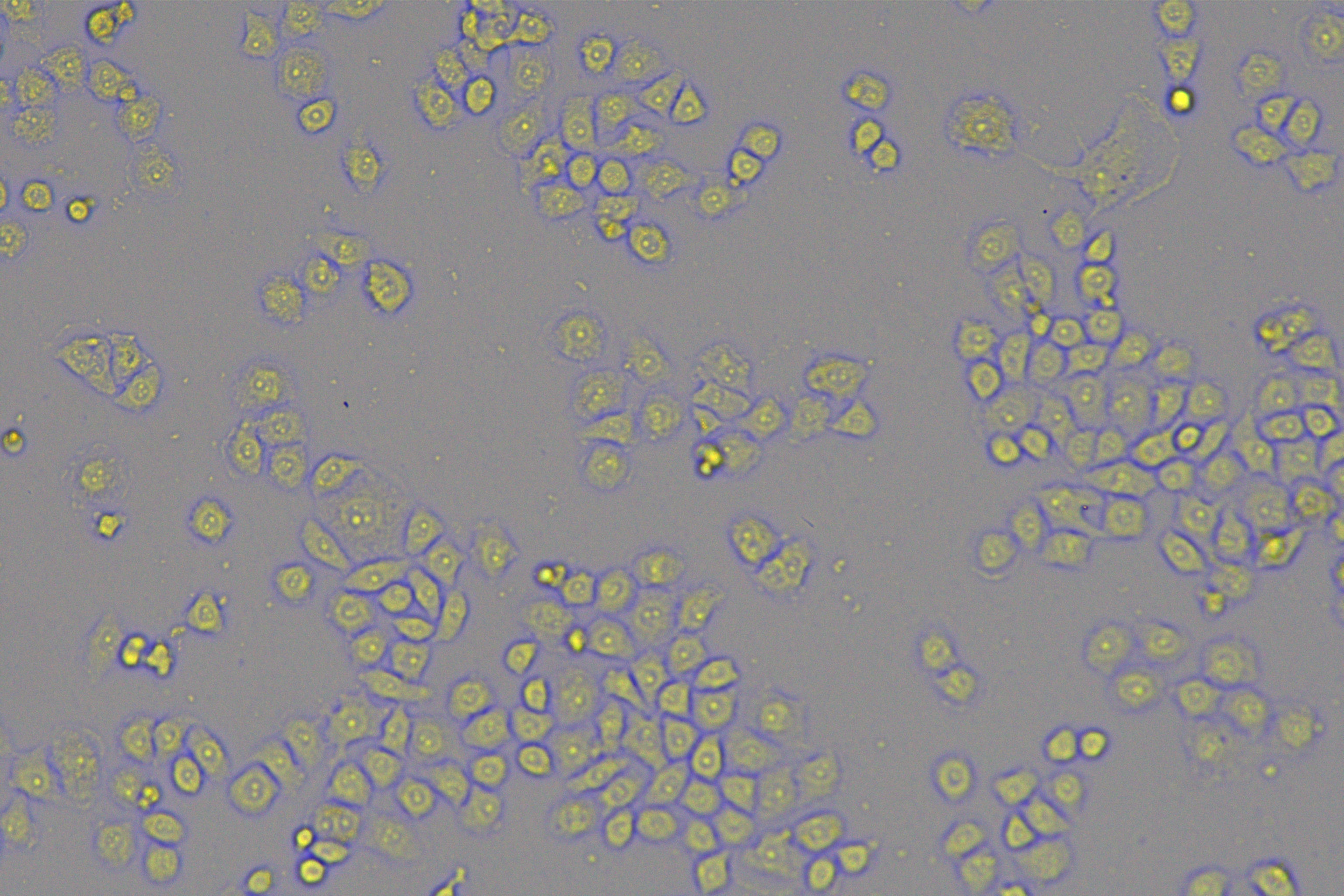

Supplement: Supplemental Information 3 [file peerj-10-12802-s003.jpg]

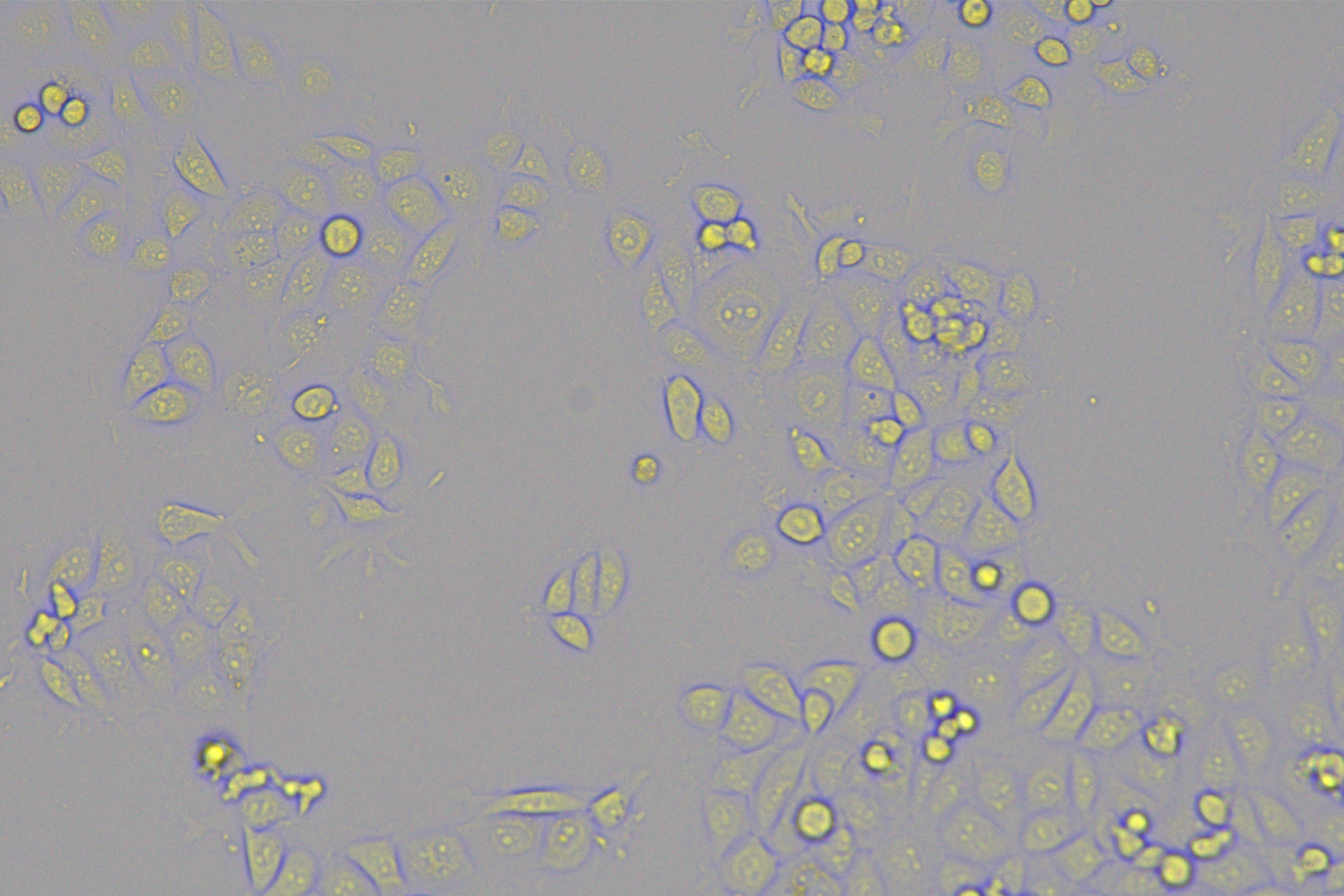

Supplement: Supplemental Information 4 [file peerj-10-12802-s004.jpg]

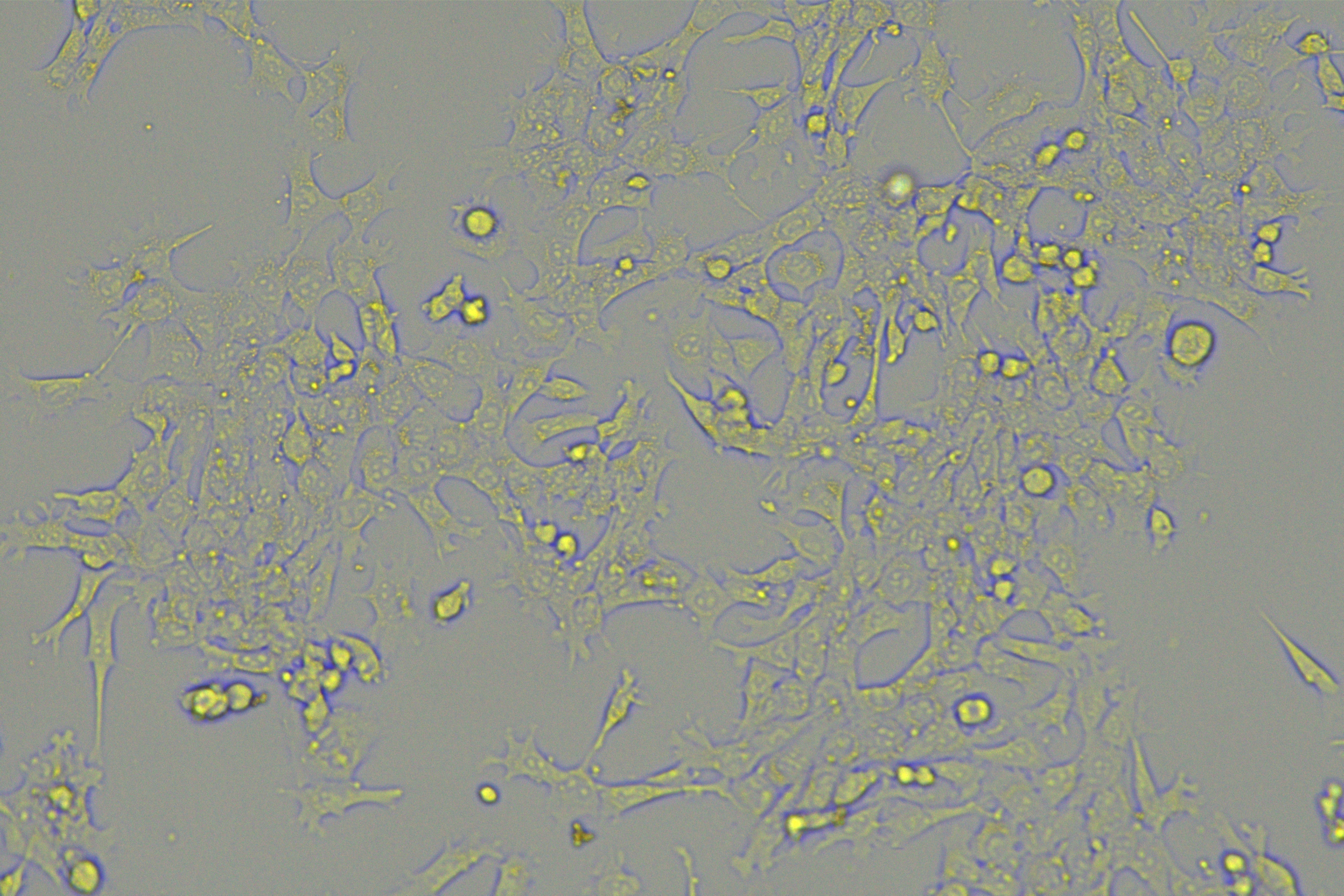

Supplement: Supplemental Information 5 [file peerj-10-12802-s005.jpg]

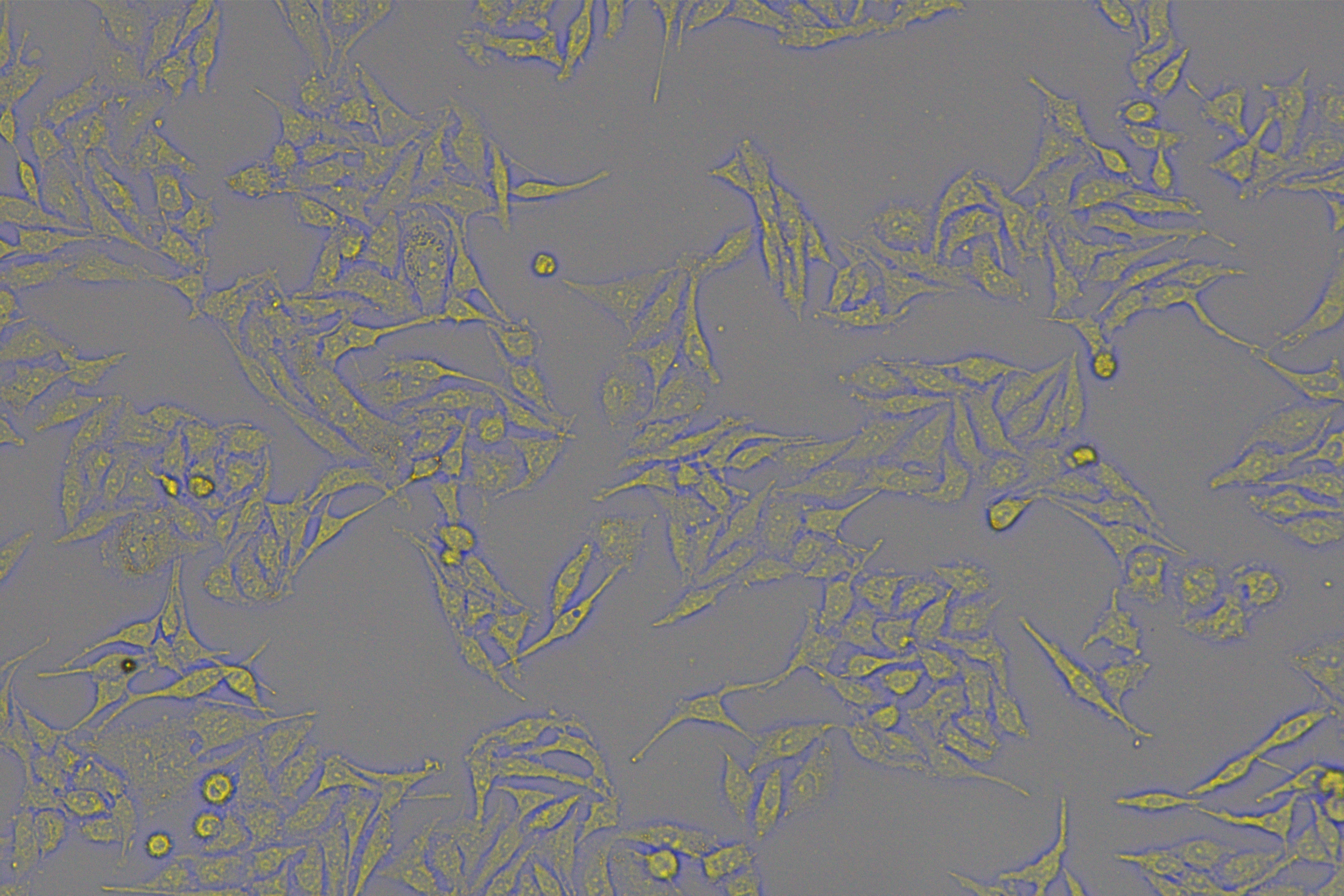

Supplement: Supplemental Information 6 [file peerj-10-12802-s006.jpg]

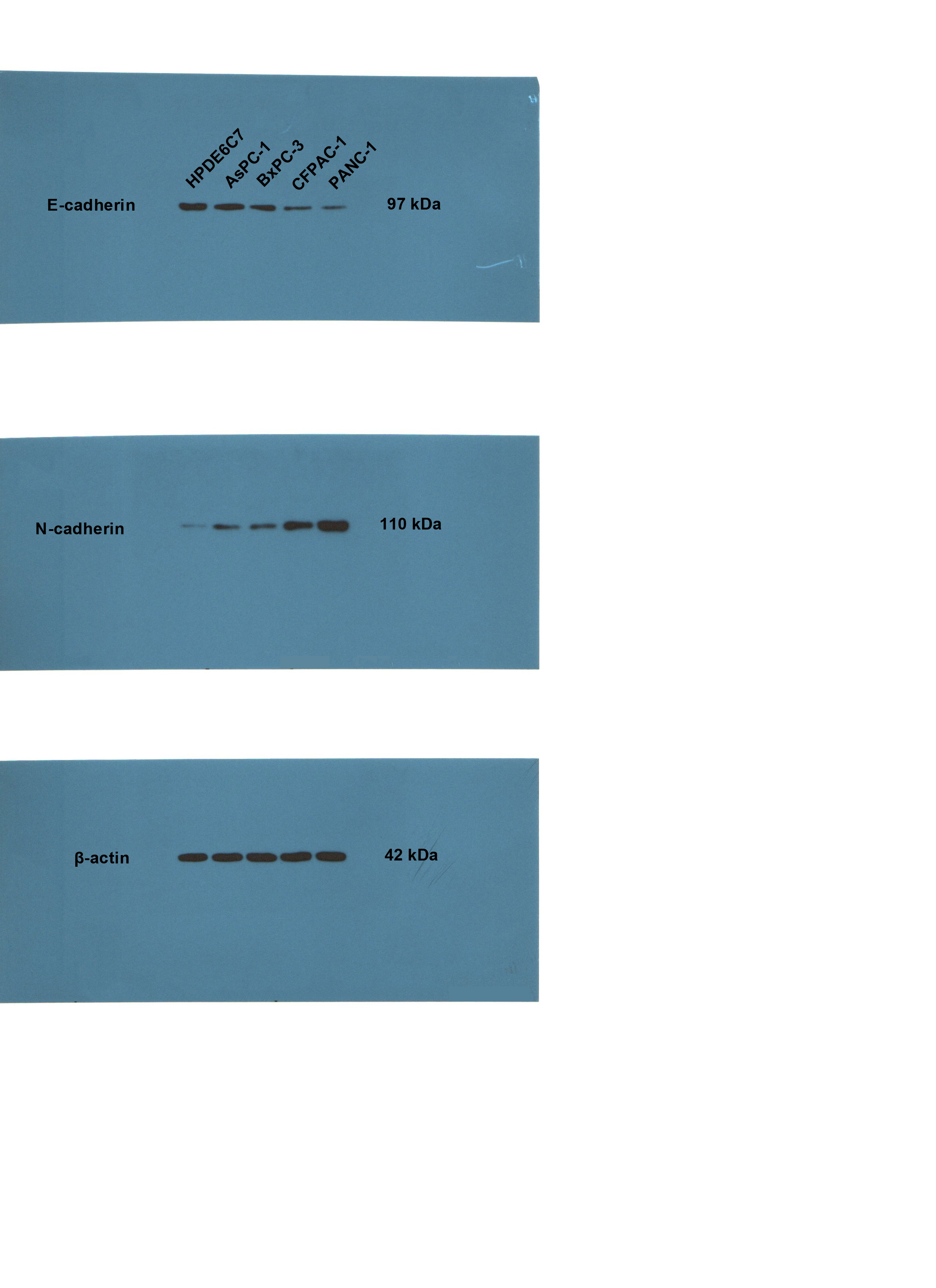

Supplement: Supplemental Information 7 [file peerj-10-12802-s007.jpg]

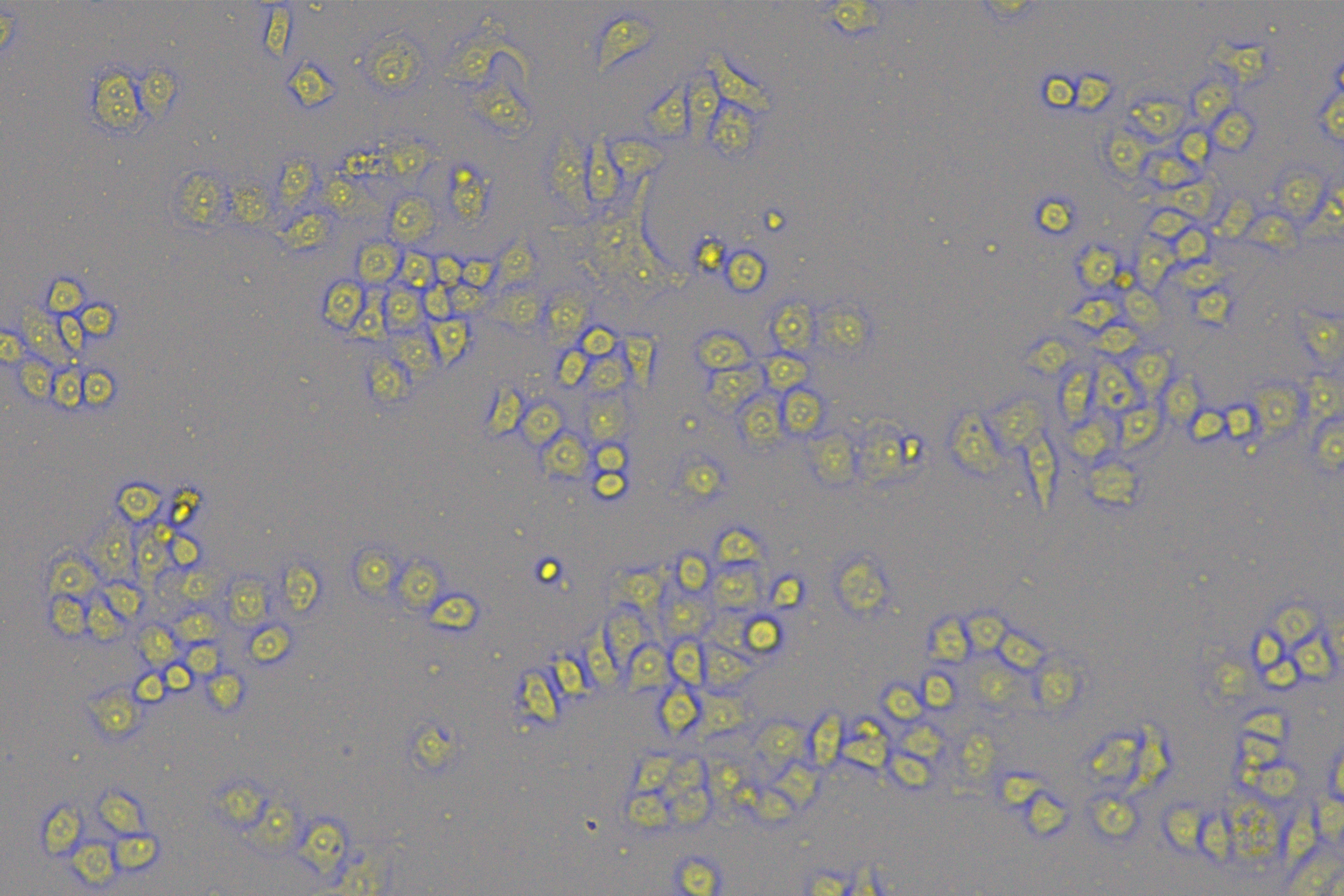

Supplement: Supplemental Information 8 [file peerj-10-12802-s008.jpg]

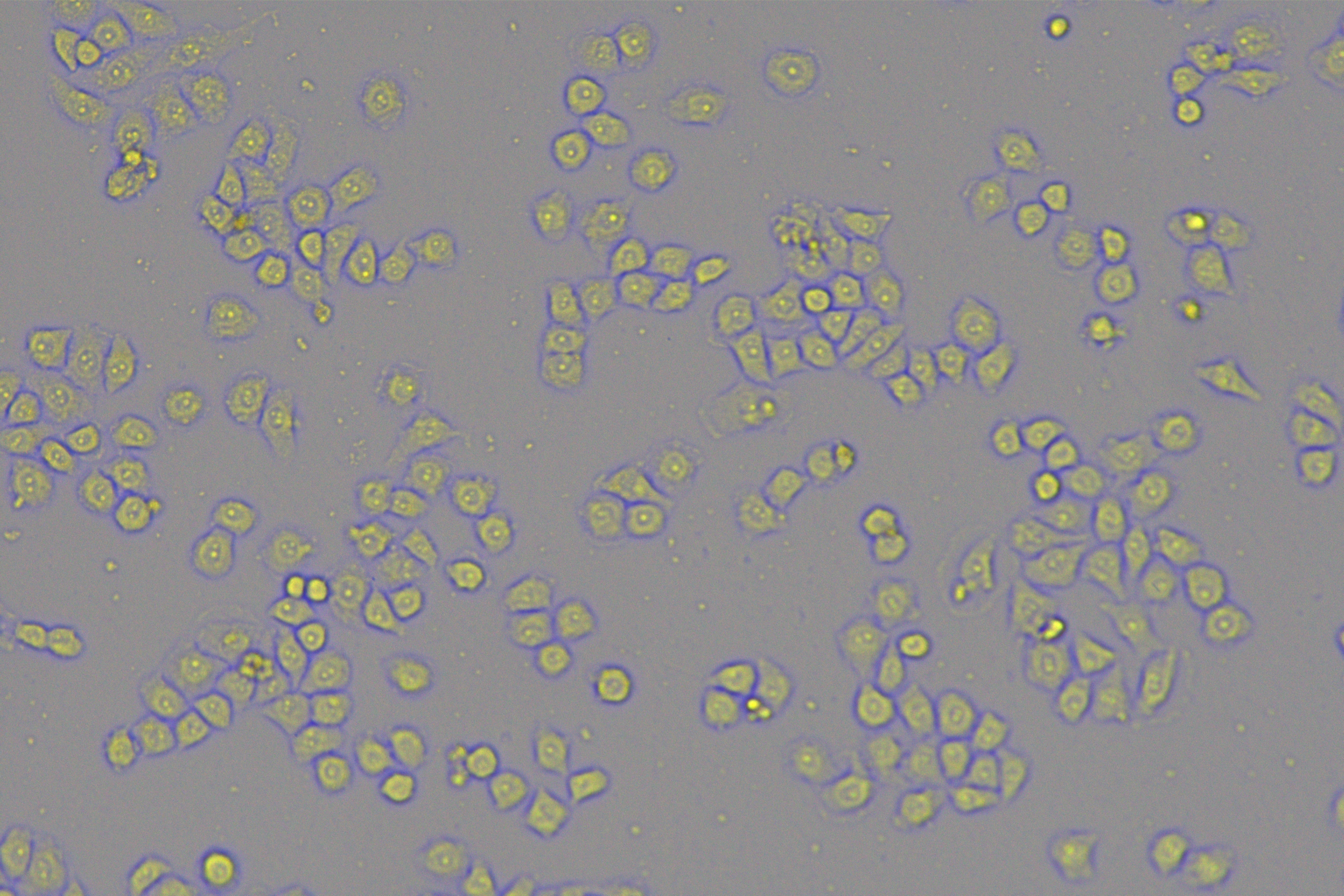

Supplement: Supplemental Information 9 [file peerj-10-12802-s009.jpg]

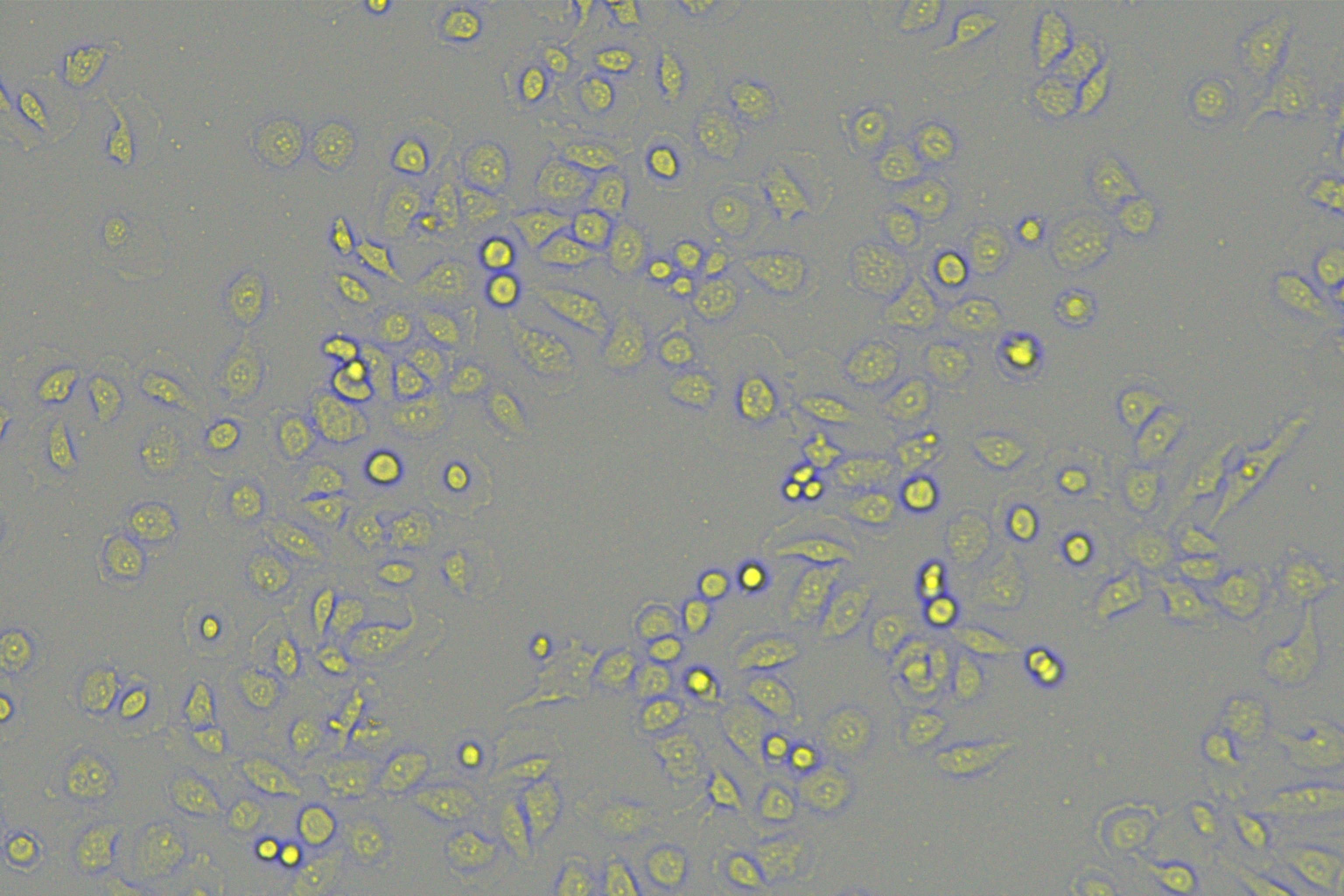

Supplement: Supplemental Information 10 [file peerj-10-12802-s010.jpg]

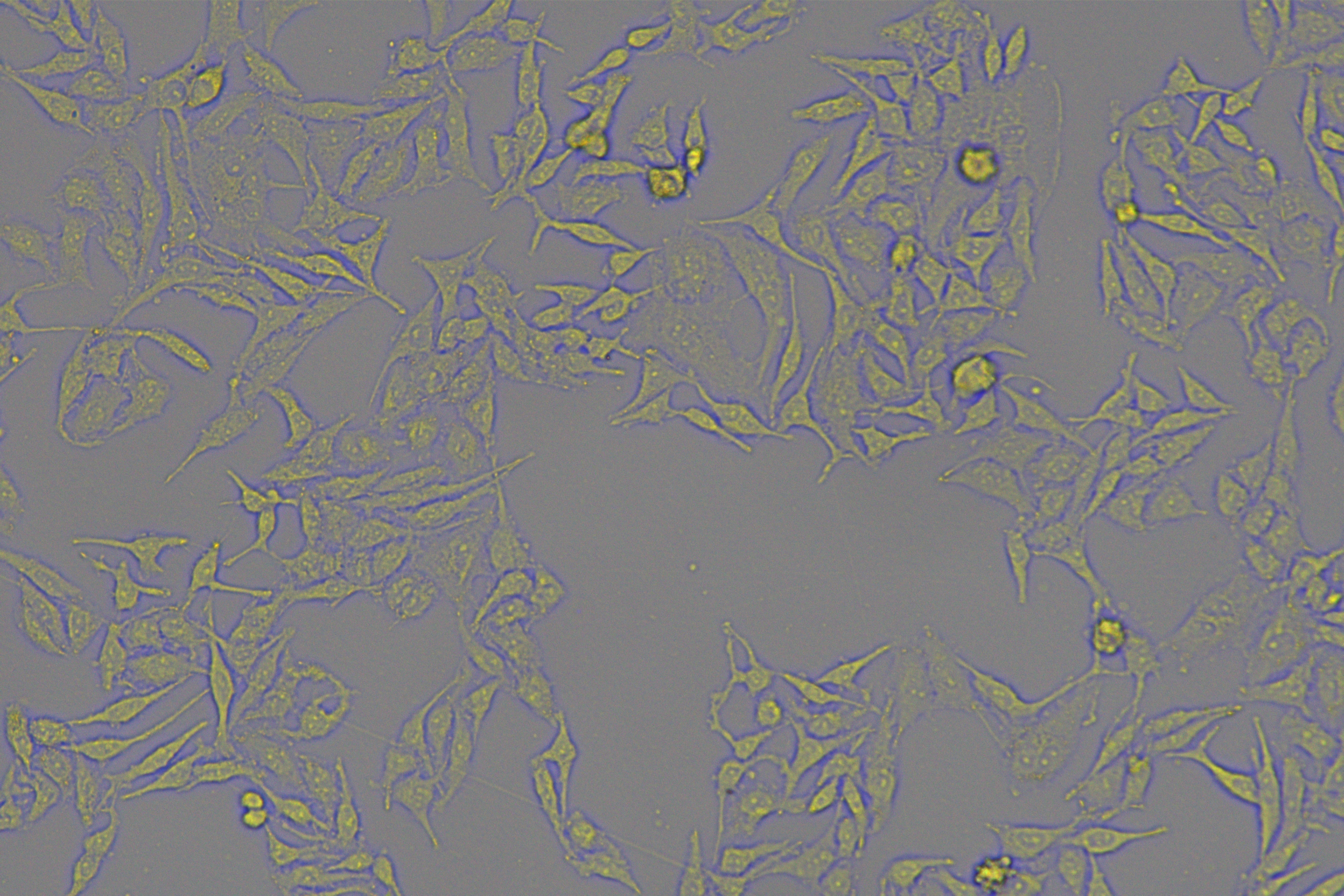

Supplement: Supplemental Information 11 [file peerj-10-12802-s011.jpg]

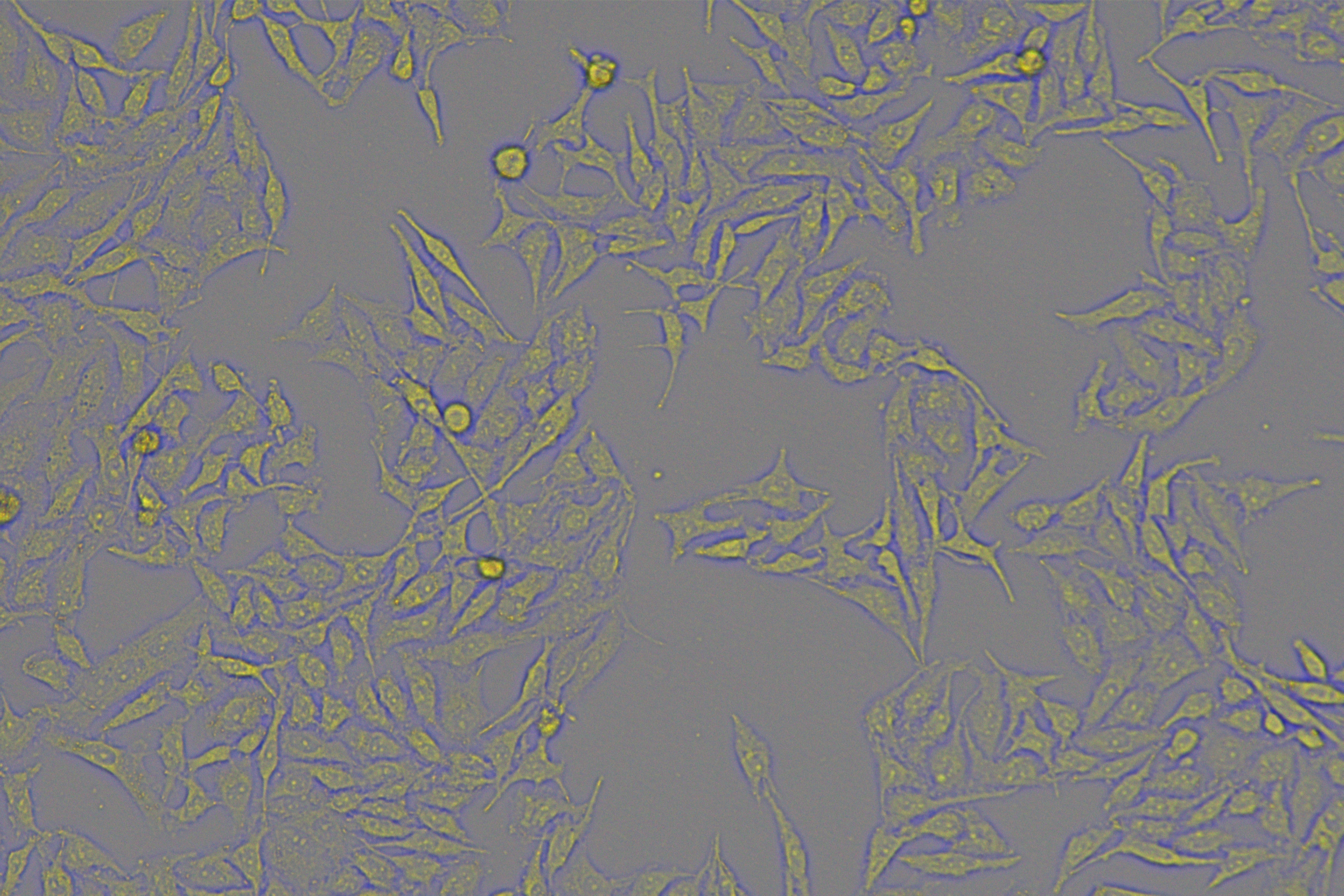

Supplement: Supplemental Information 12 [file peerj-10-12802-s012.jpg]

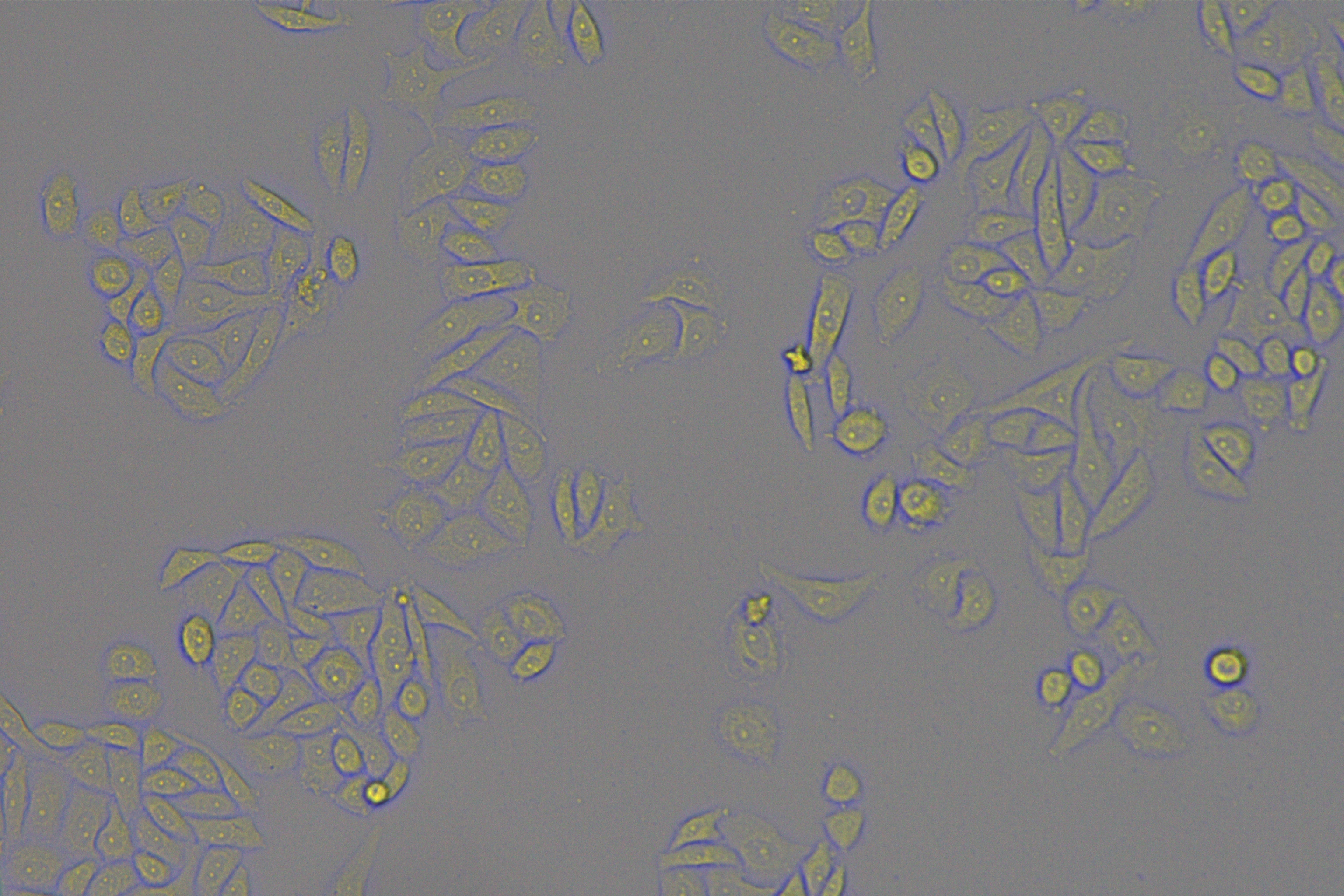

Supplement: Supplemental Information 13 [file peerj-10-12802-s013.jpg]

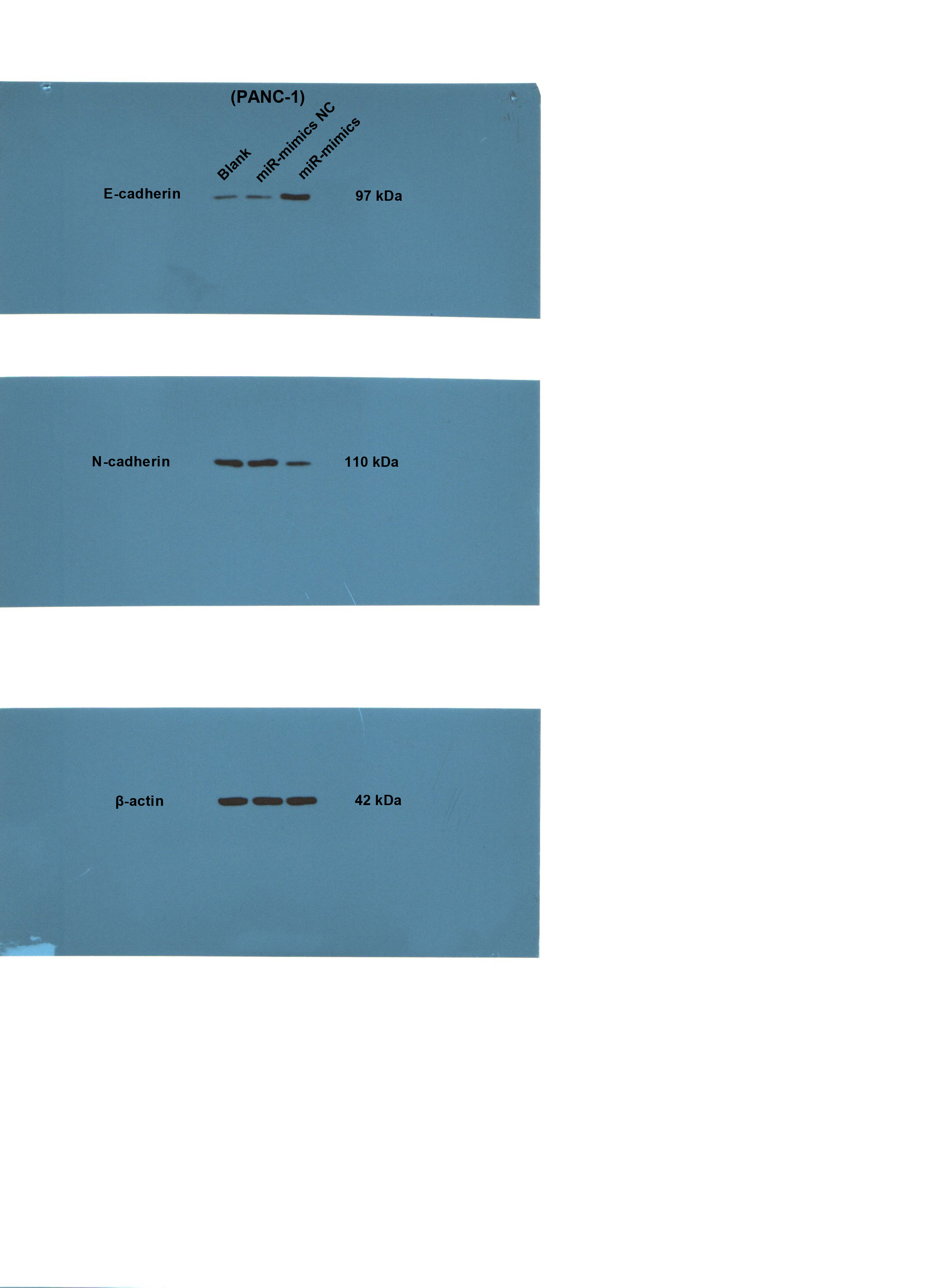

Supplement: Supplemental Information 14 [file peerj-10-12802-s014.jpg]

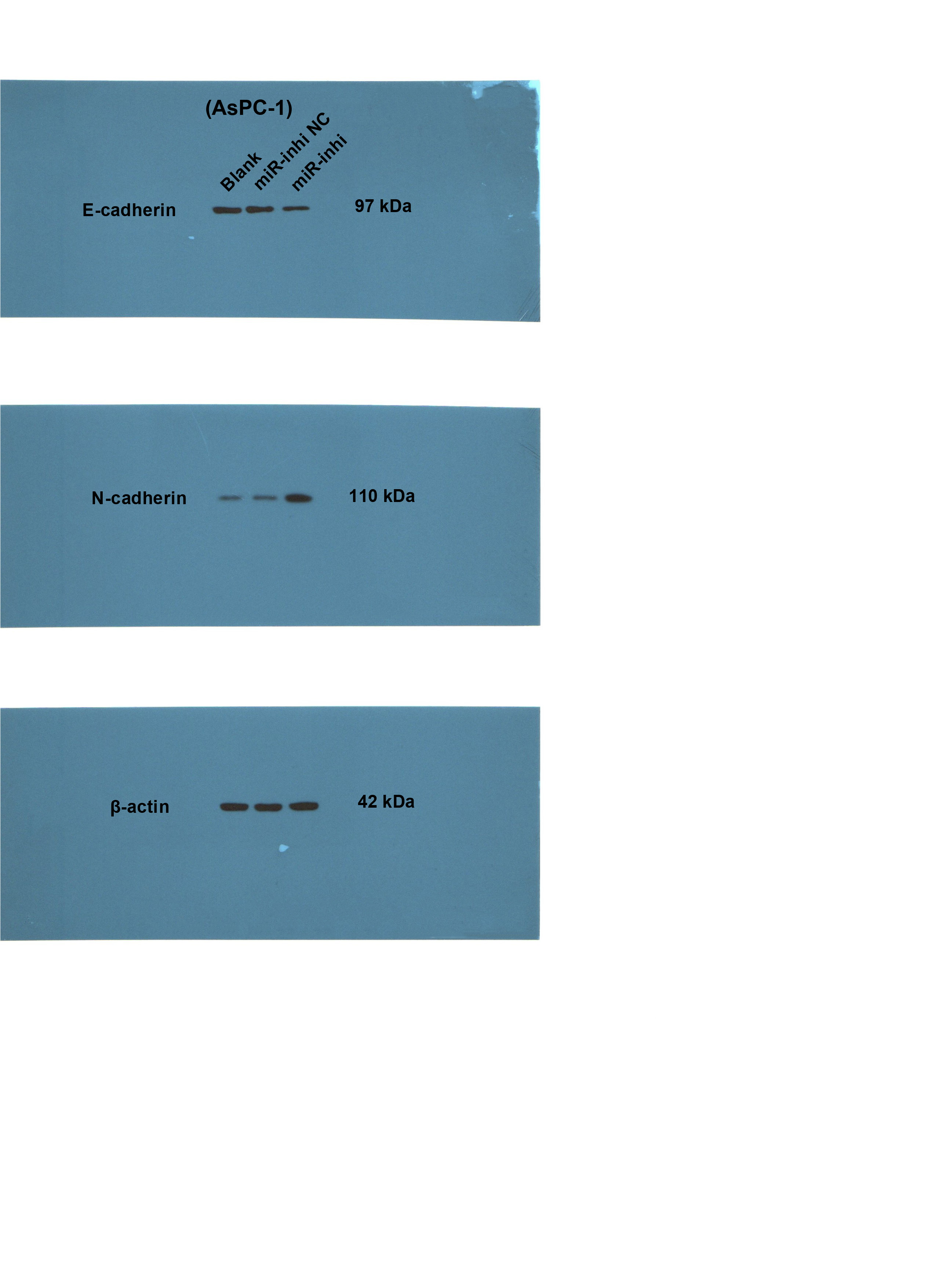

Supplement: Supplemental Information 15 [file peerj-10-12802-s015.jpg]

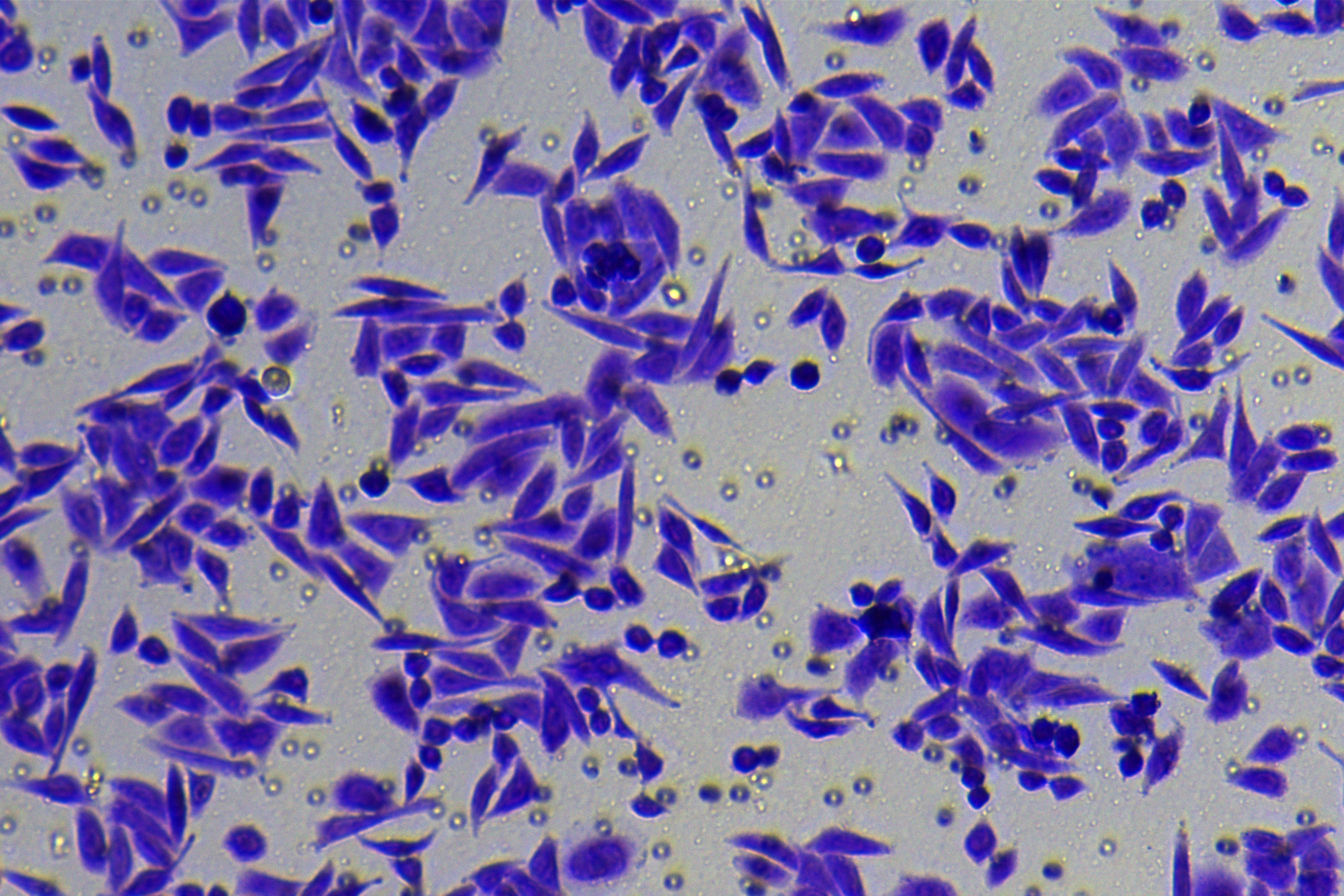

Supplement: Supplemental Information 16 [file peerj-10-12802-s016.jpg]

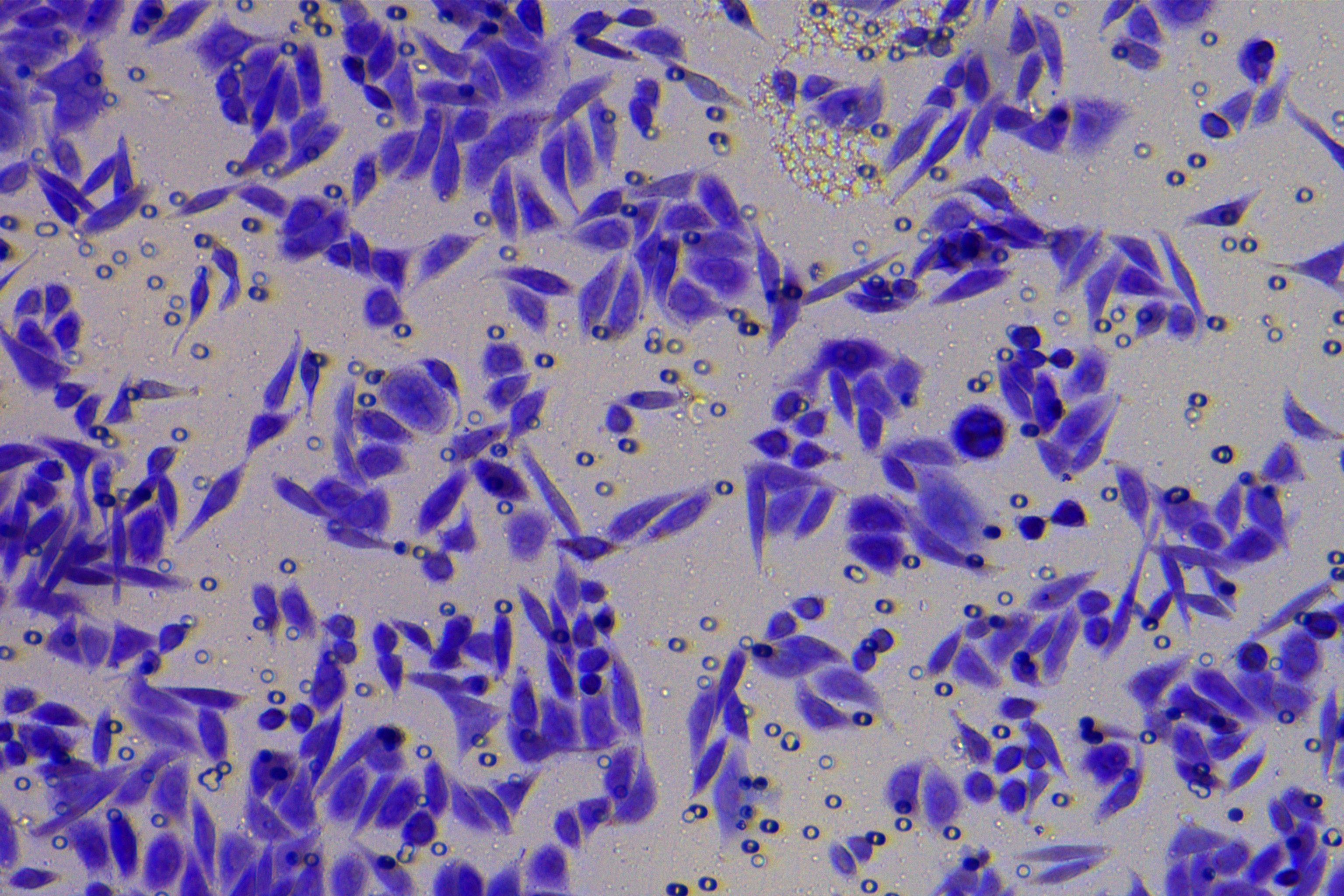

Supplement: Supplemental Information 17 [file peerj-10-12802-s017.jpg]

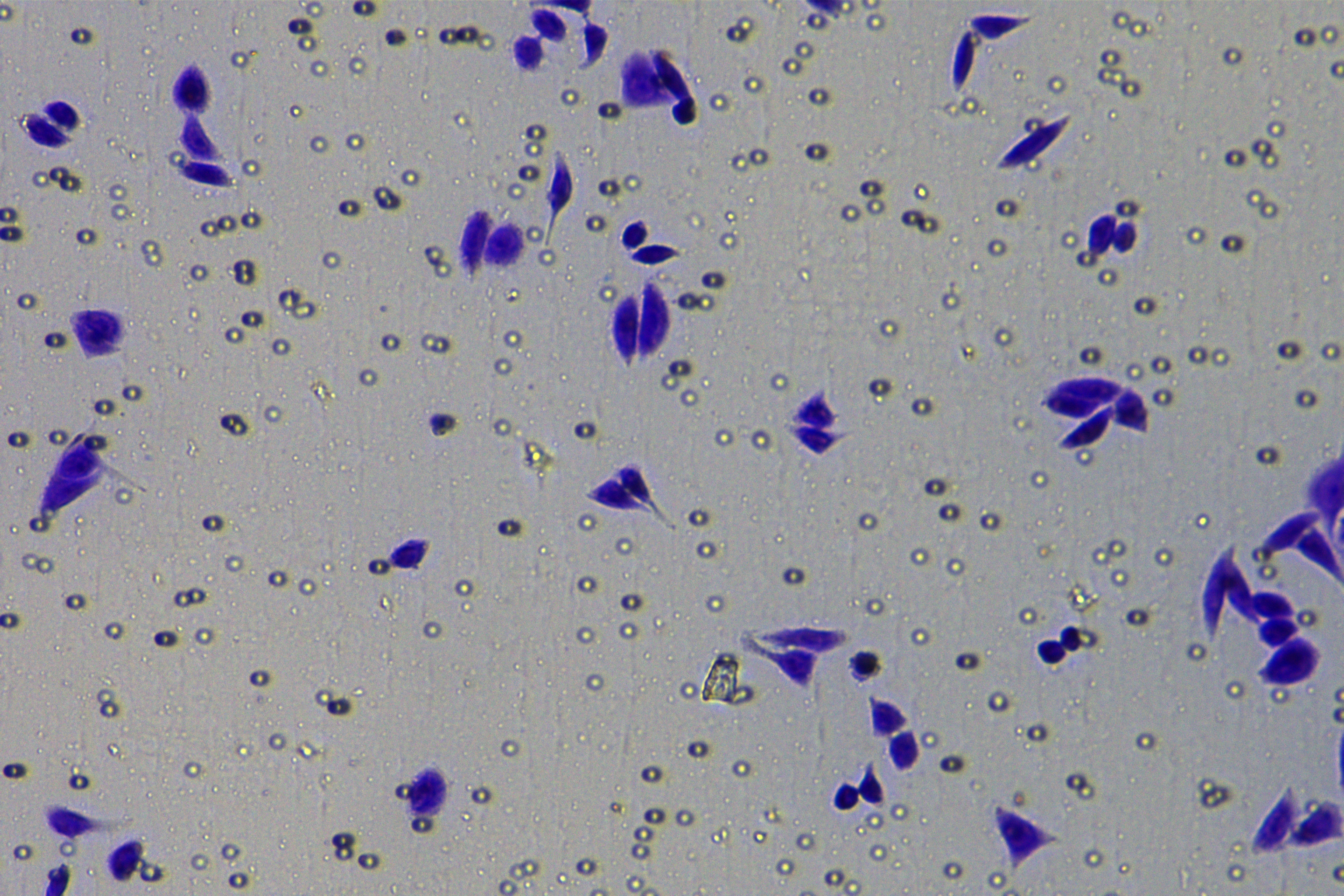

Supplement: Supplemental Information 18 [file peerj-10-12802-s018.jpg]

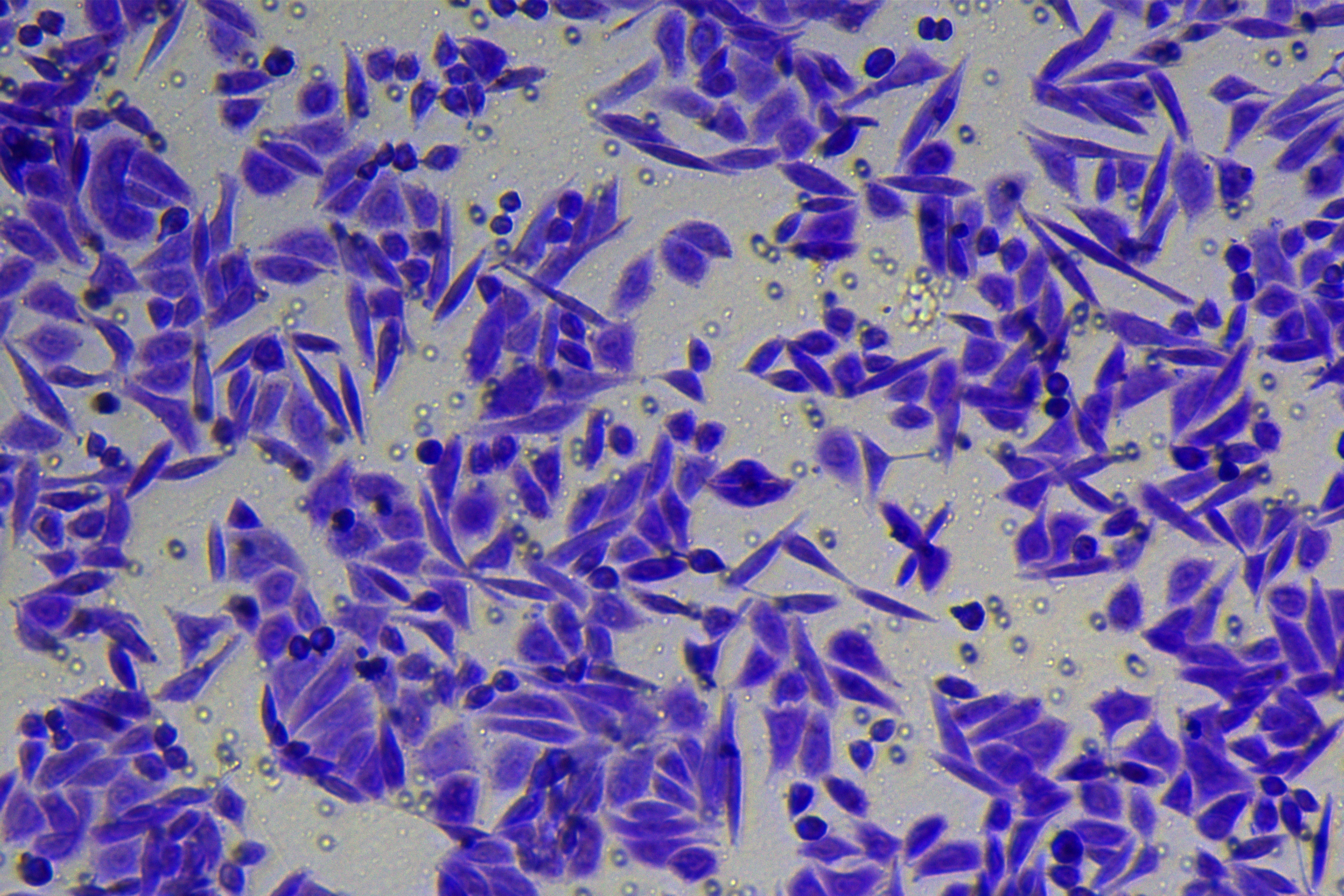

Supplement: Supplemental Information 19 [file peerj-10-12802-s019.jpg]

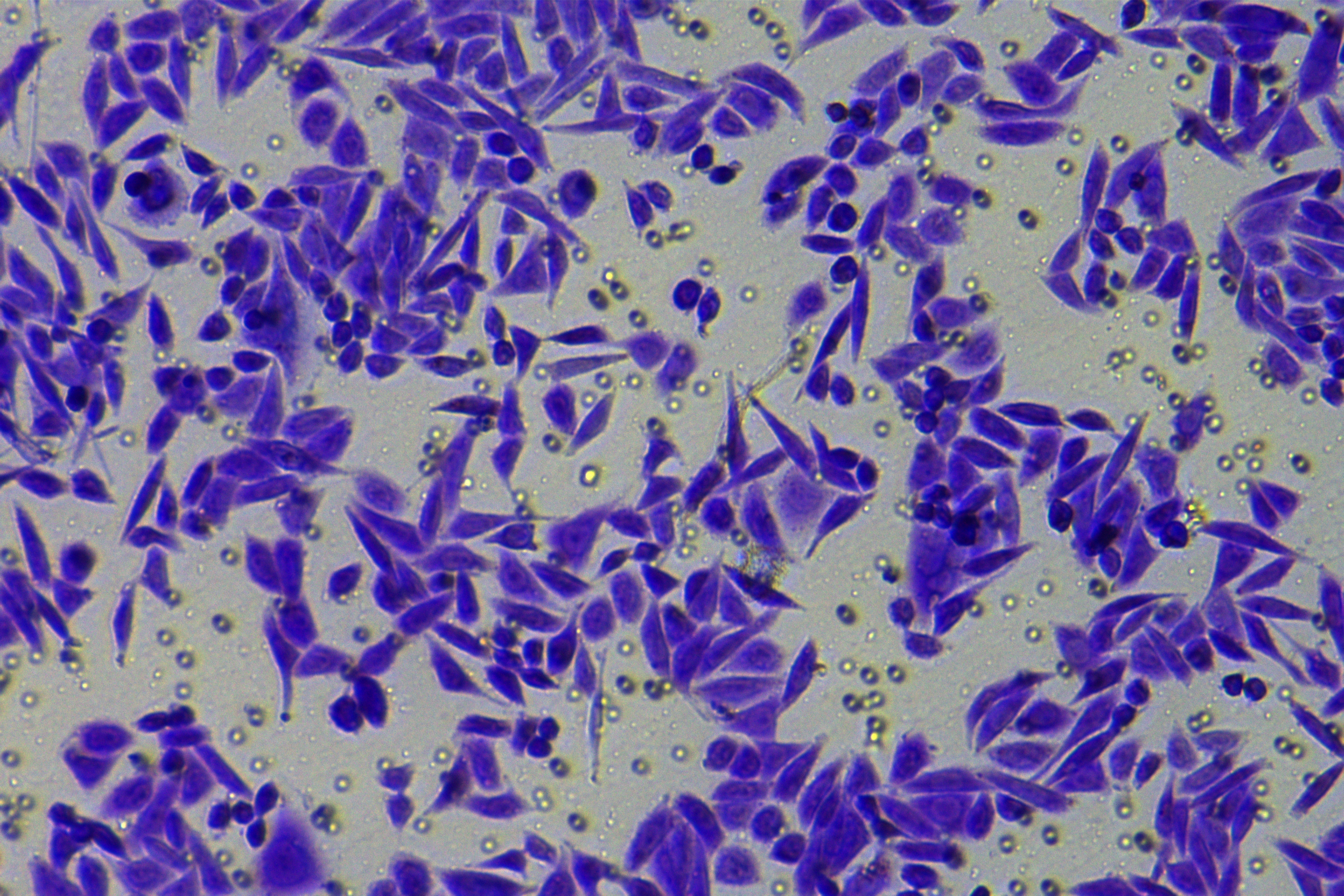

Supplement: Supplemental Information 20 [file peerj-10-12802-s020.jpg]

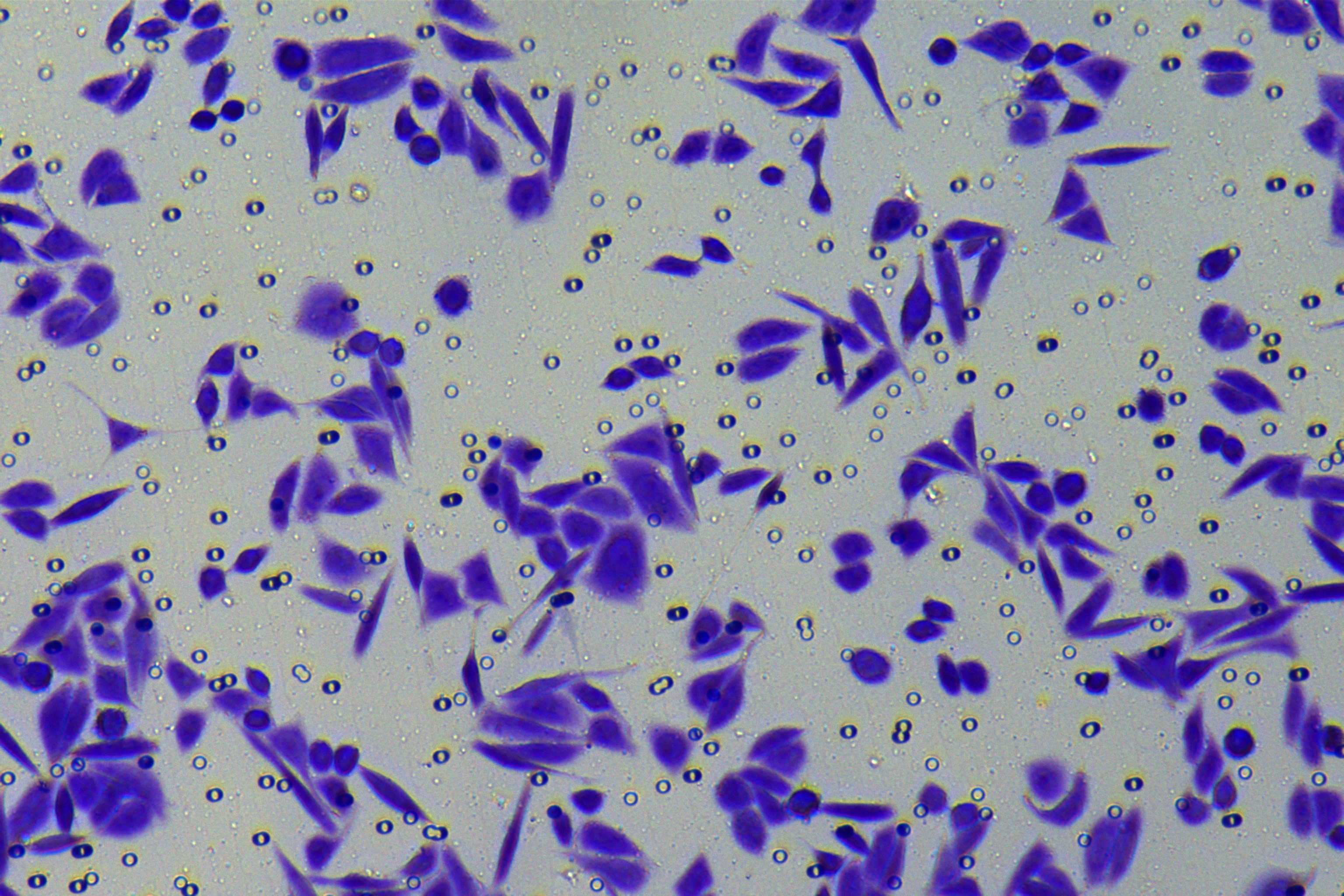

Supplement: Supplemental Information 21 [file peerj-10-12802-s021.jpg]

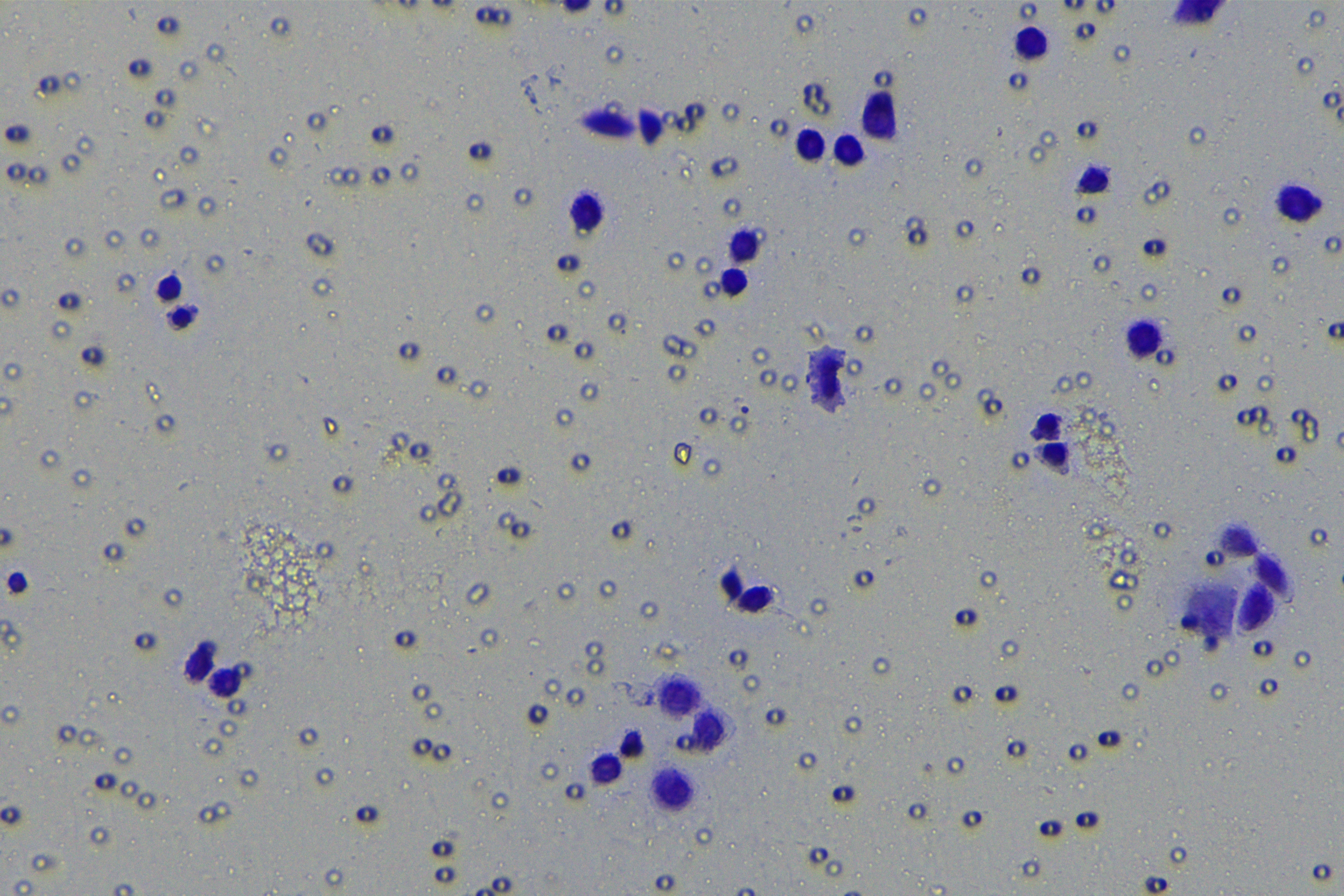

Supplement: Supplemental Information 22 [file peerj-10-12802-s022.jpg]

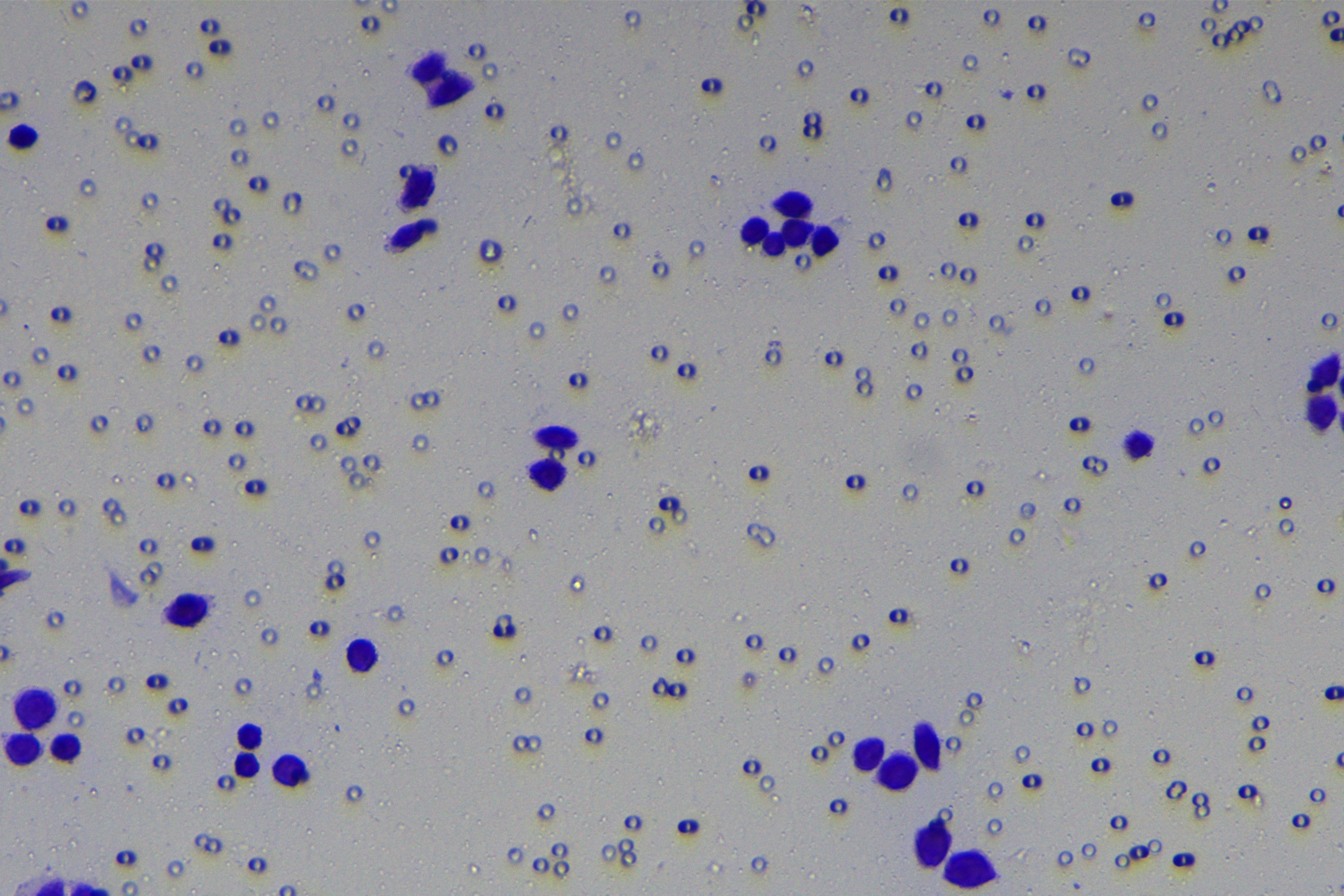

Supplement: Supplemental Information 23 [file peerj-10-12802-s023.jpg]

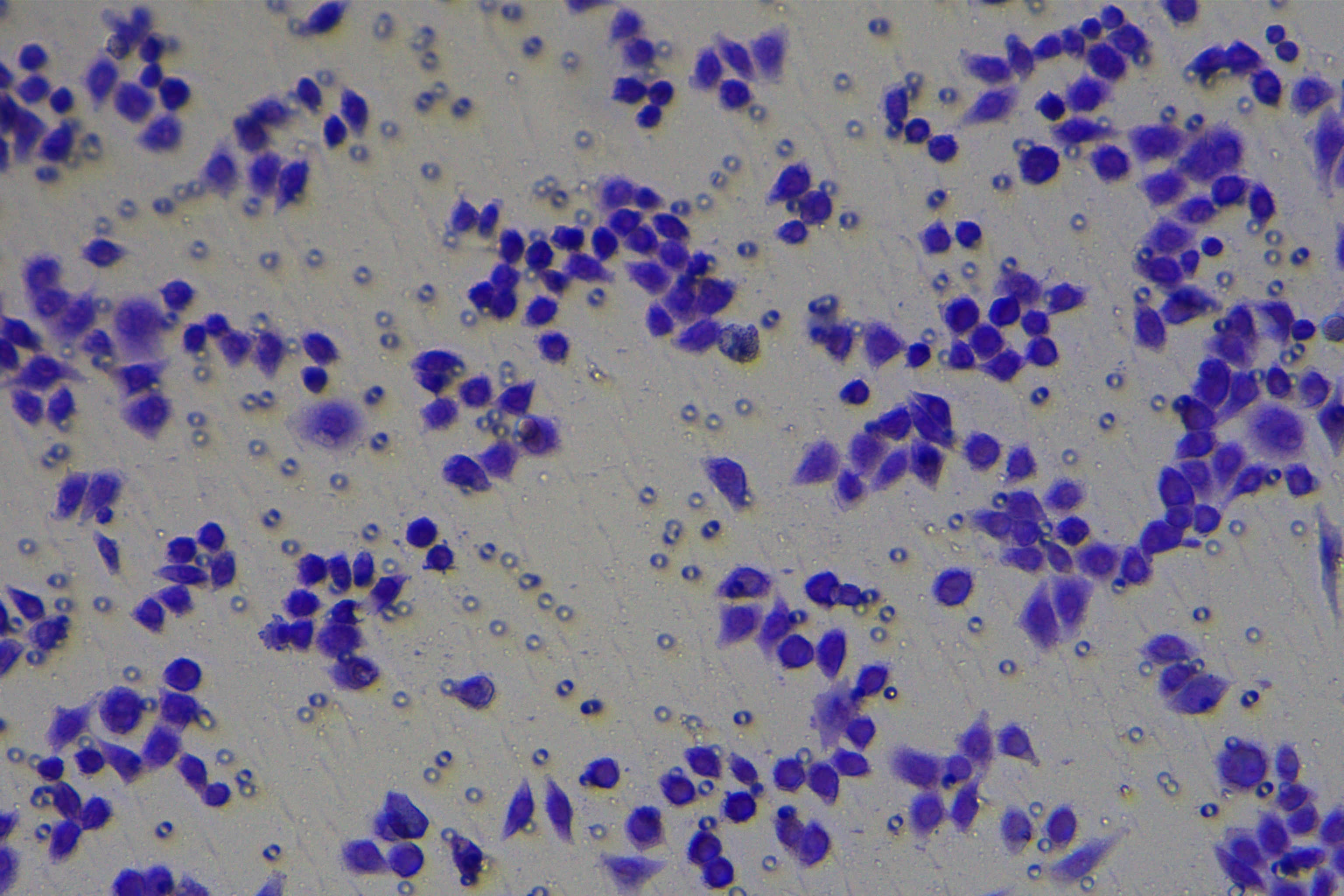

Supplement: Supplemental Information 24 [file peerj-10-12802-s024.jpg]

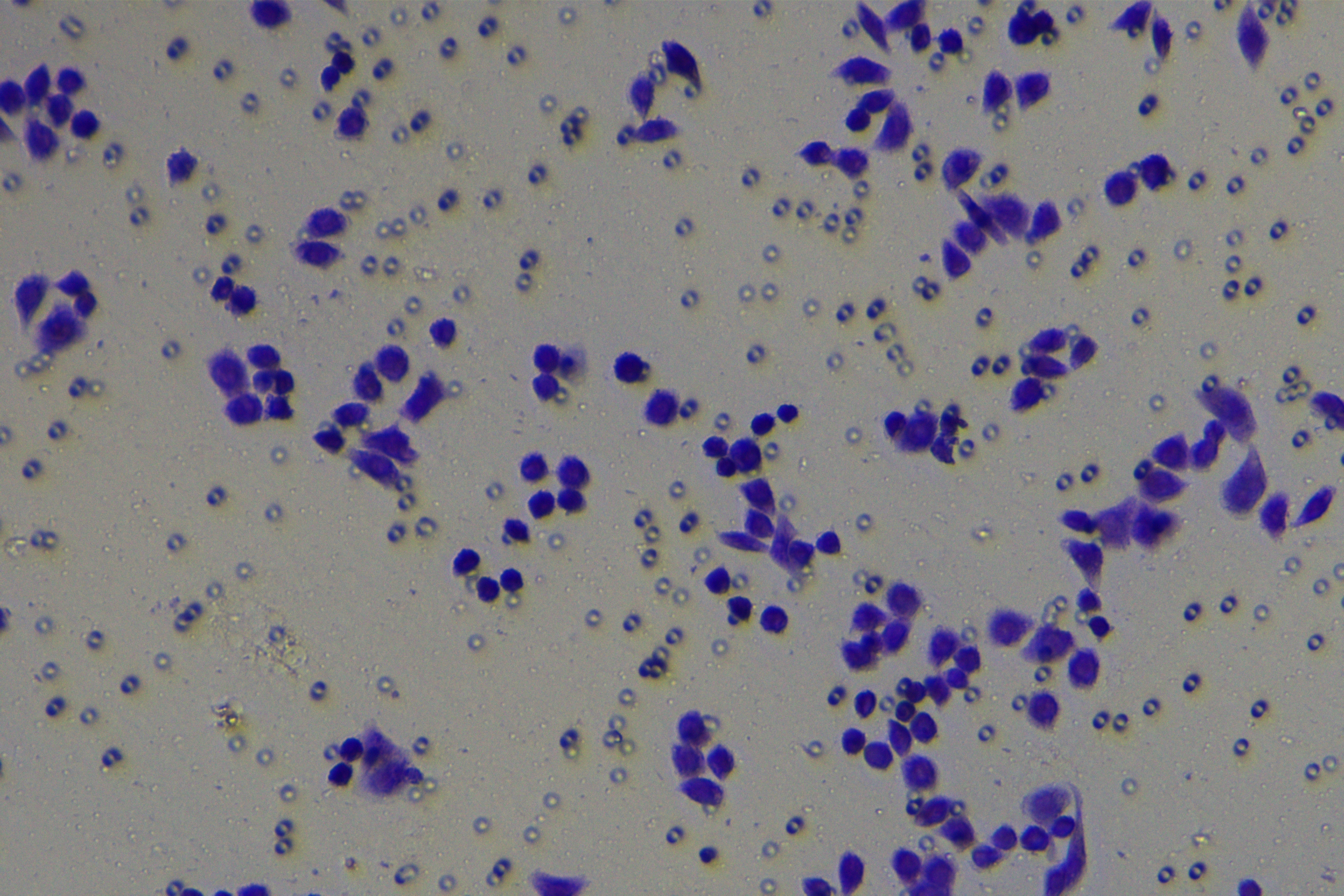

Supplement: Supplemental Information 25 [file peerj-10-12802-s025.jpg]

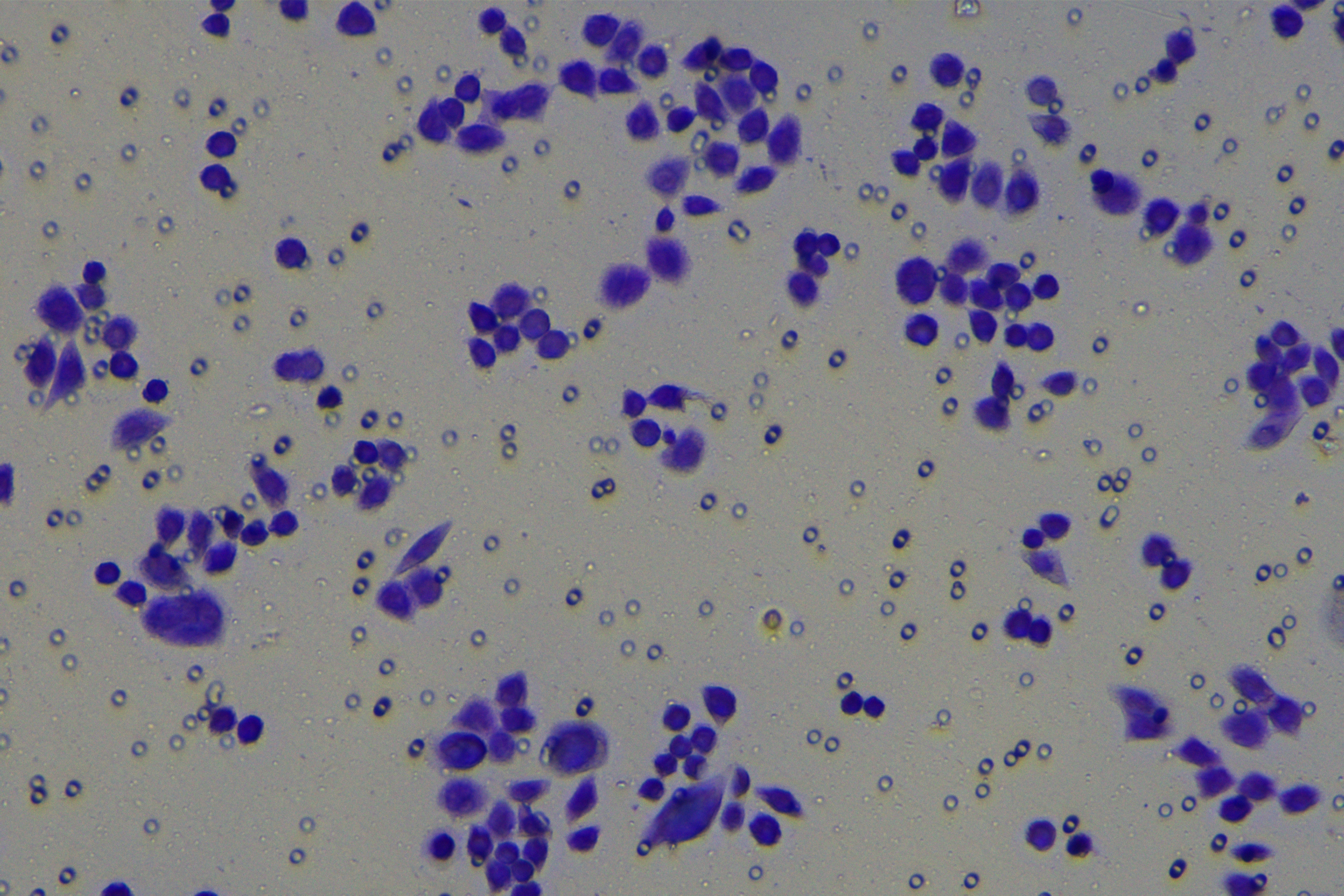

Supplement: Supplemental Information 26 [file peerj-10-12802-s026.jpg]

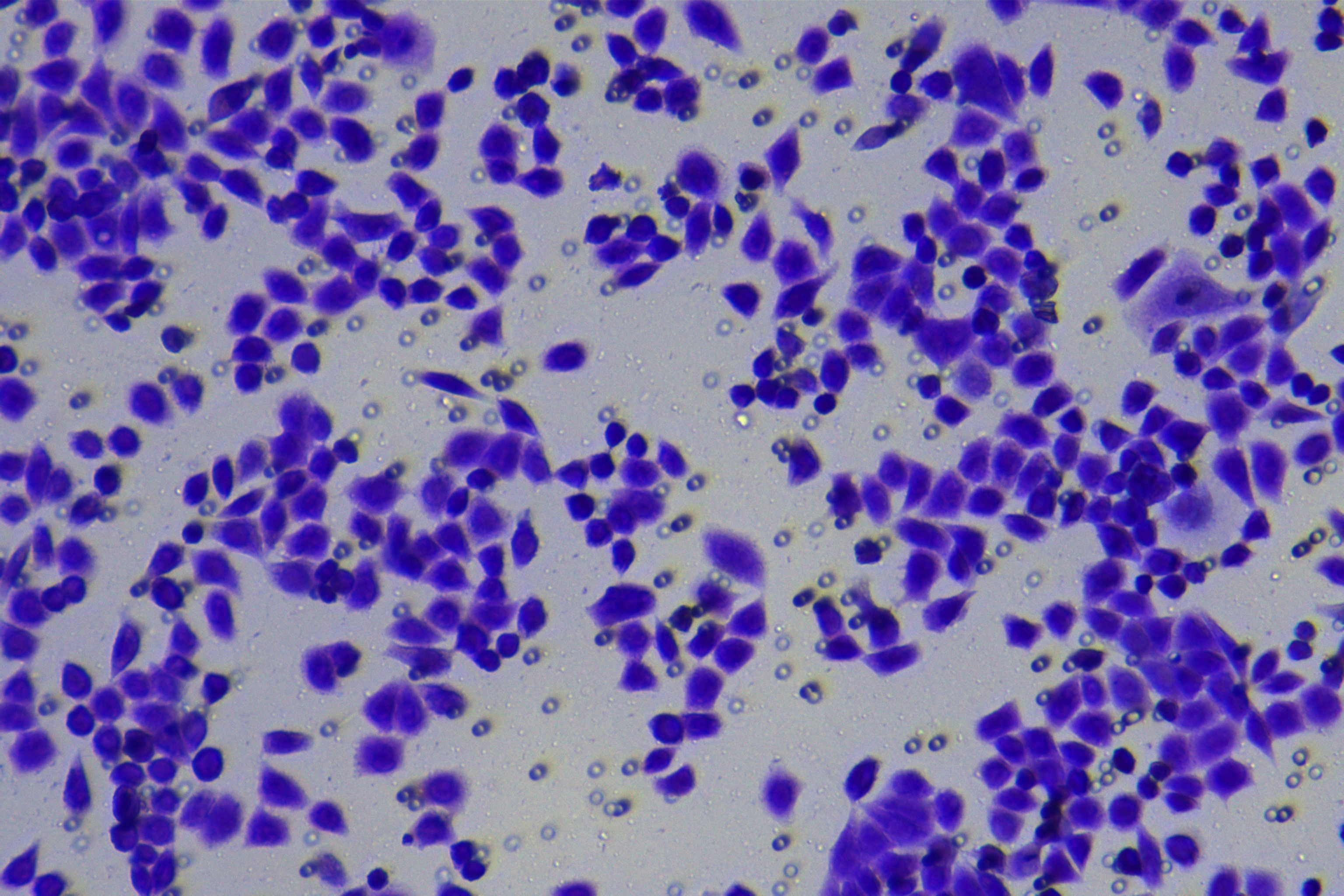

Supplement: Supplemental Information 27 [file peerj-10-12802-s027.jpg]

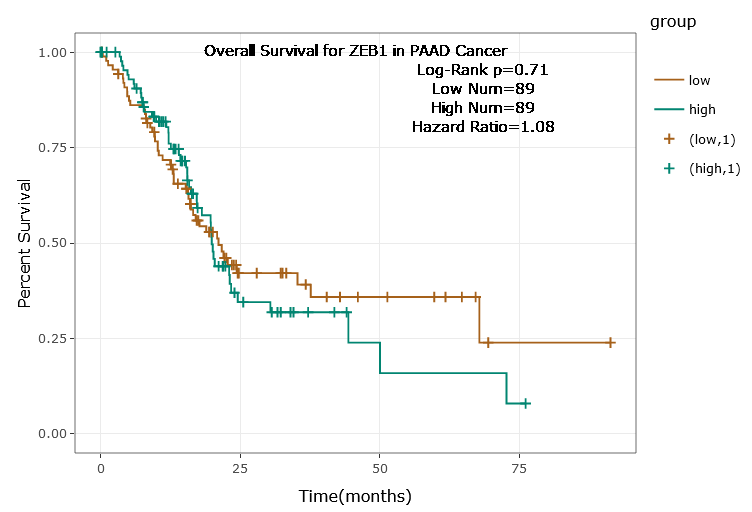

Supplement: Supplemental Information 28 [file peerj-10-12802-s028.png]

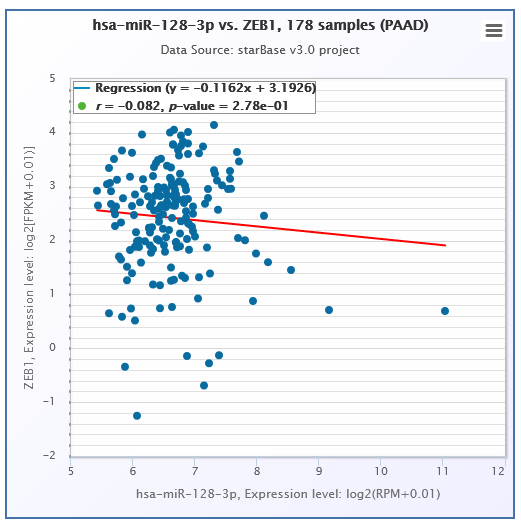

Supplement: Supplemental Information 29 [file peerj-10-12802-s029.png]

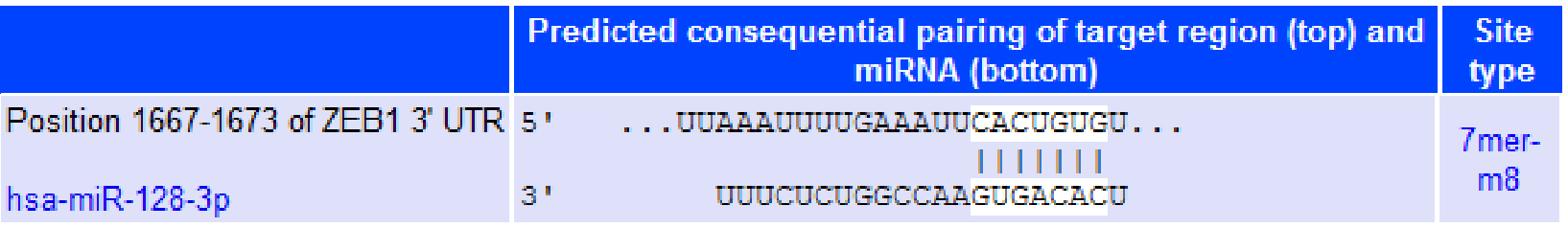

Supplement: Supplemental Information 30 [file peerj-10-12802-s030.jpg]

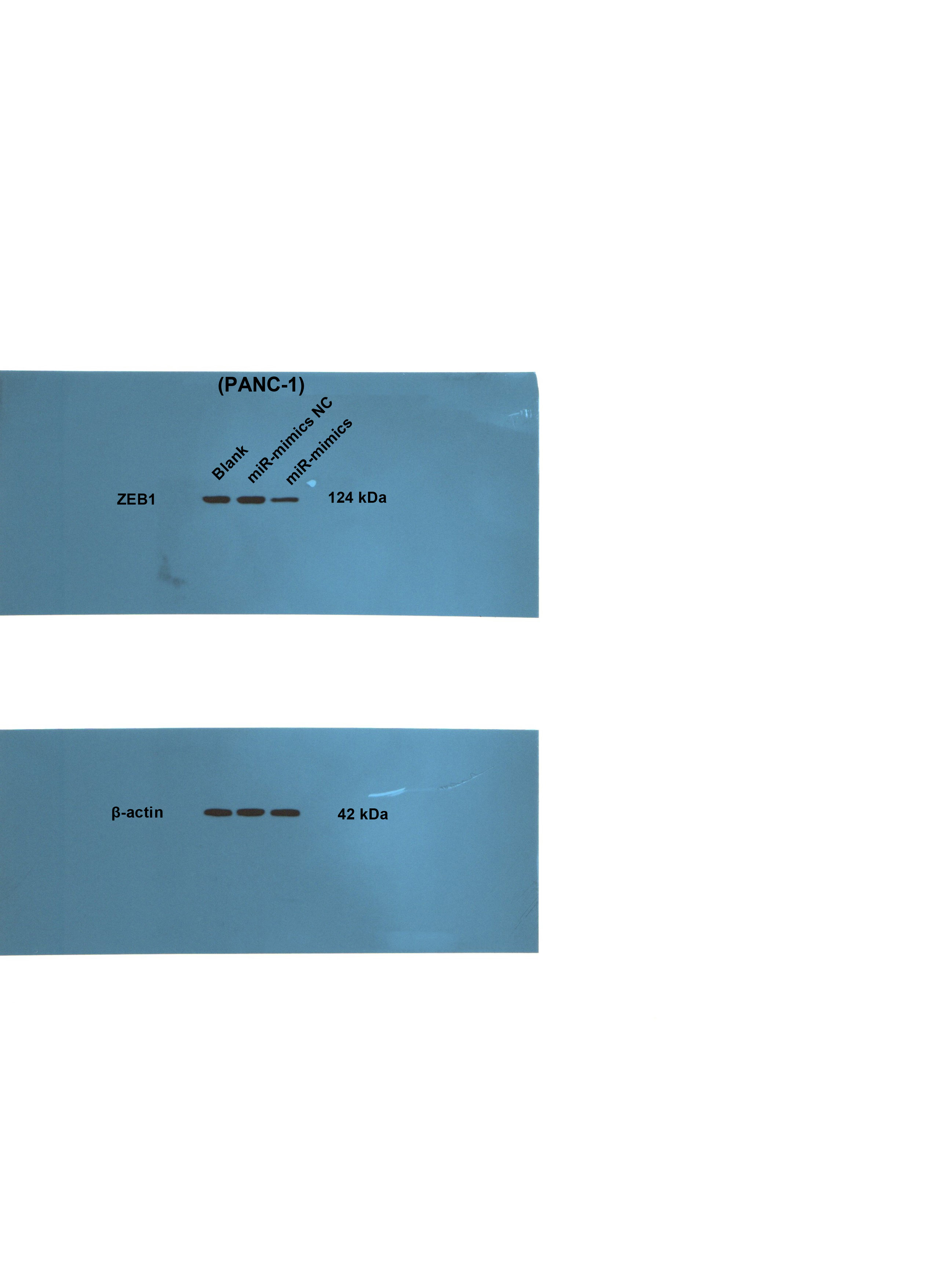

Supplement: Supplemental Information 31 [file peerj-10-12802-s031.jpg]

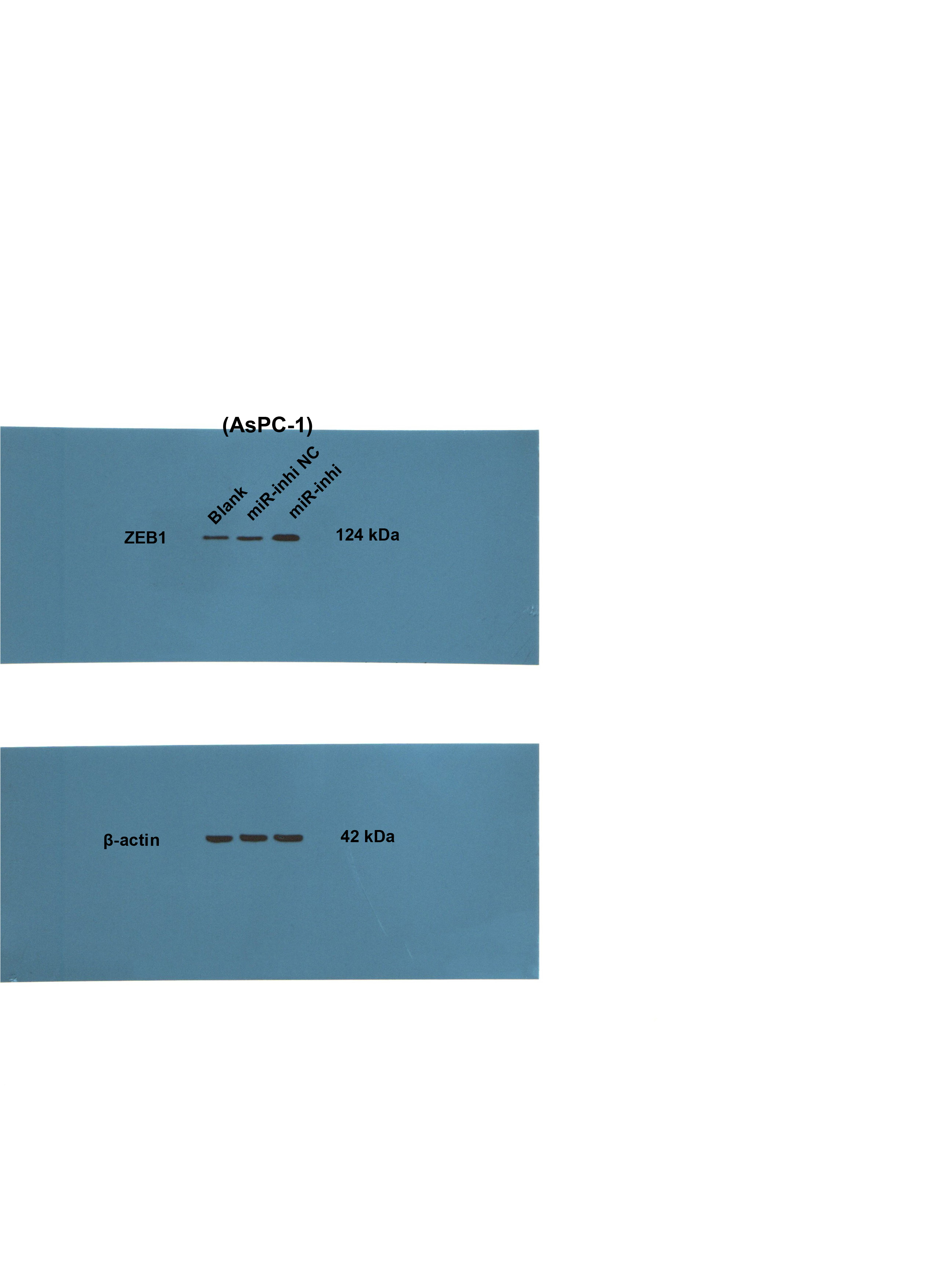

Supplement: Supplemental Information 32 [file peerj-10-12802-s032.jpg]

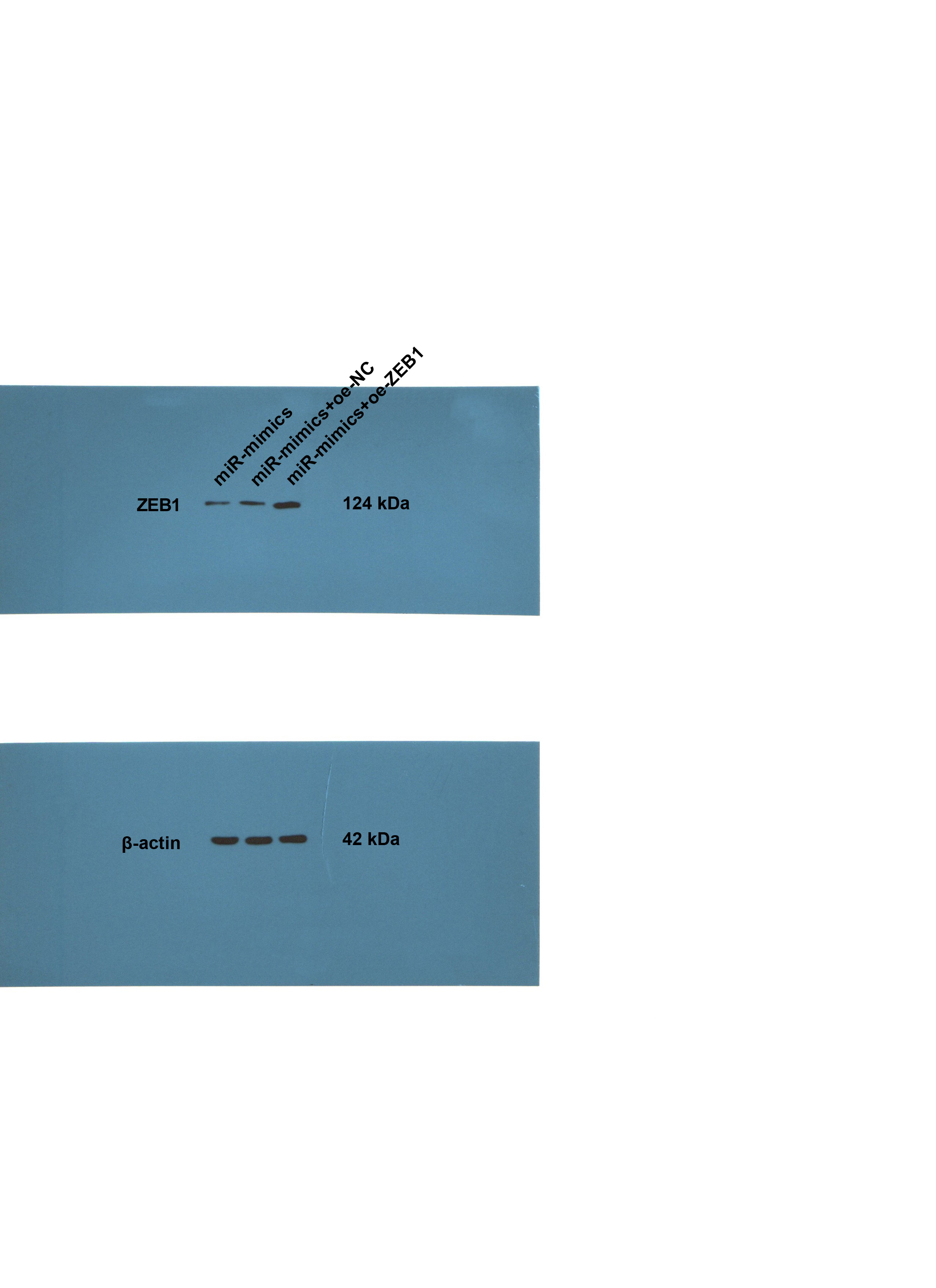

Supplement: Supplemental Information 33 [file peerj-10-12802-s033.jpg]

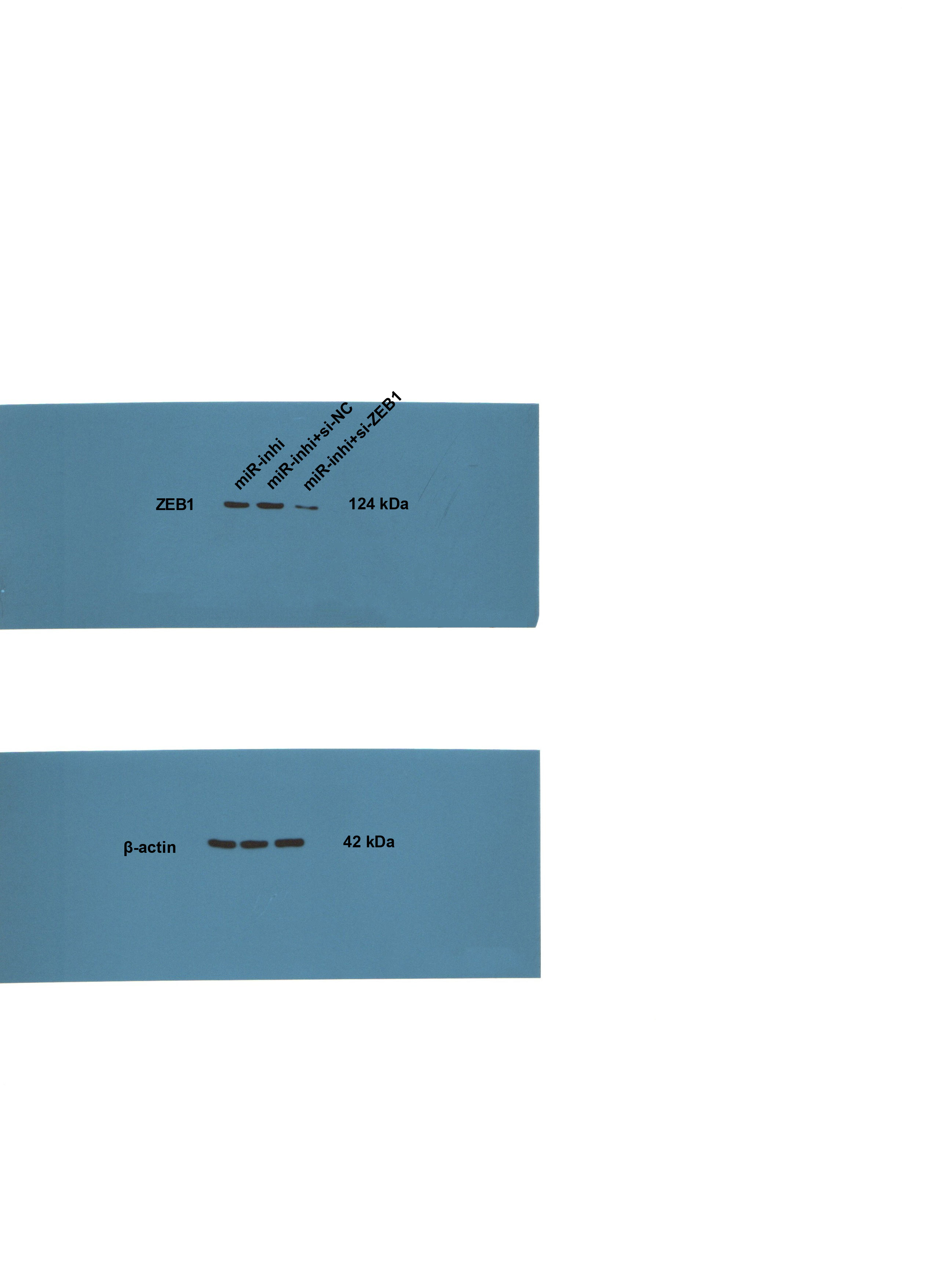

Supplement: Supplemental Information 34 [file peerj-10-12802-s034.jpg]

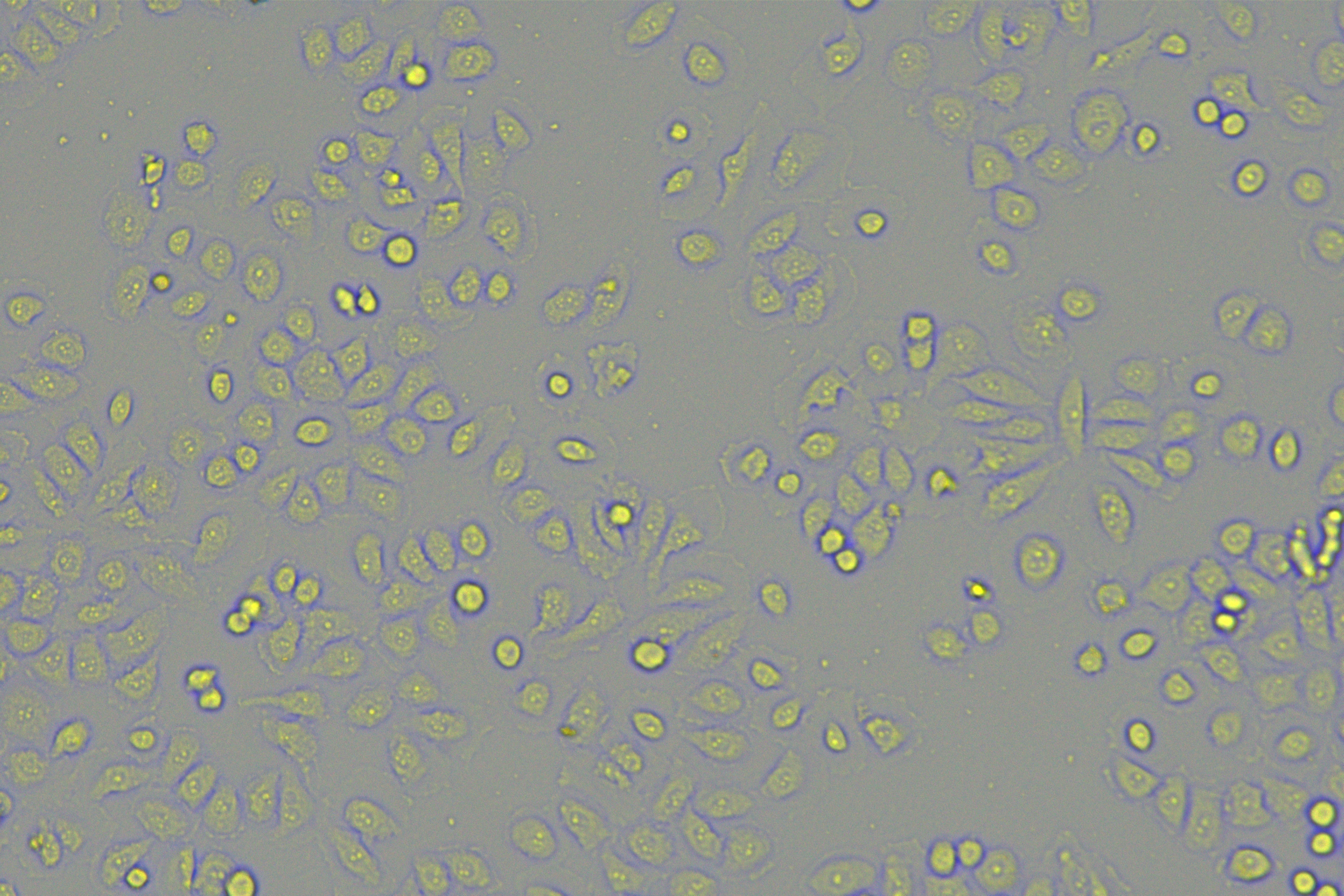

Supplement: Supplemental Information 35 [file peerj-10-12802-s035.jpg]

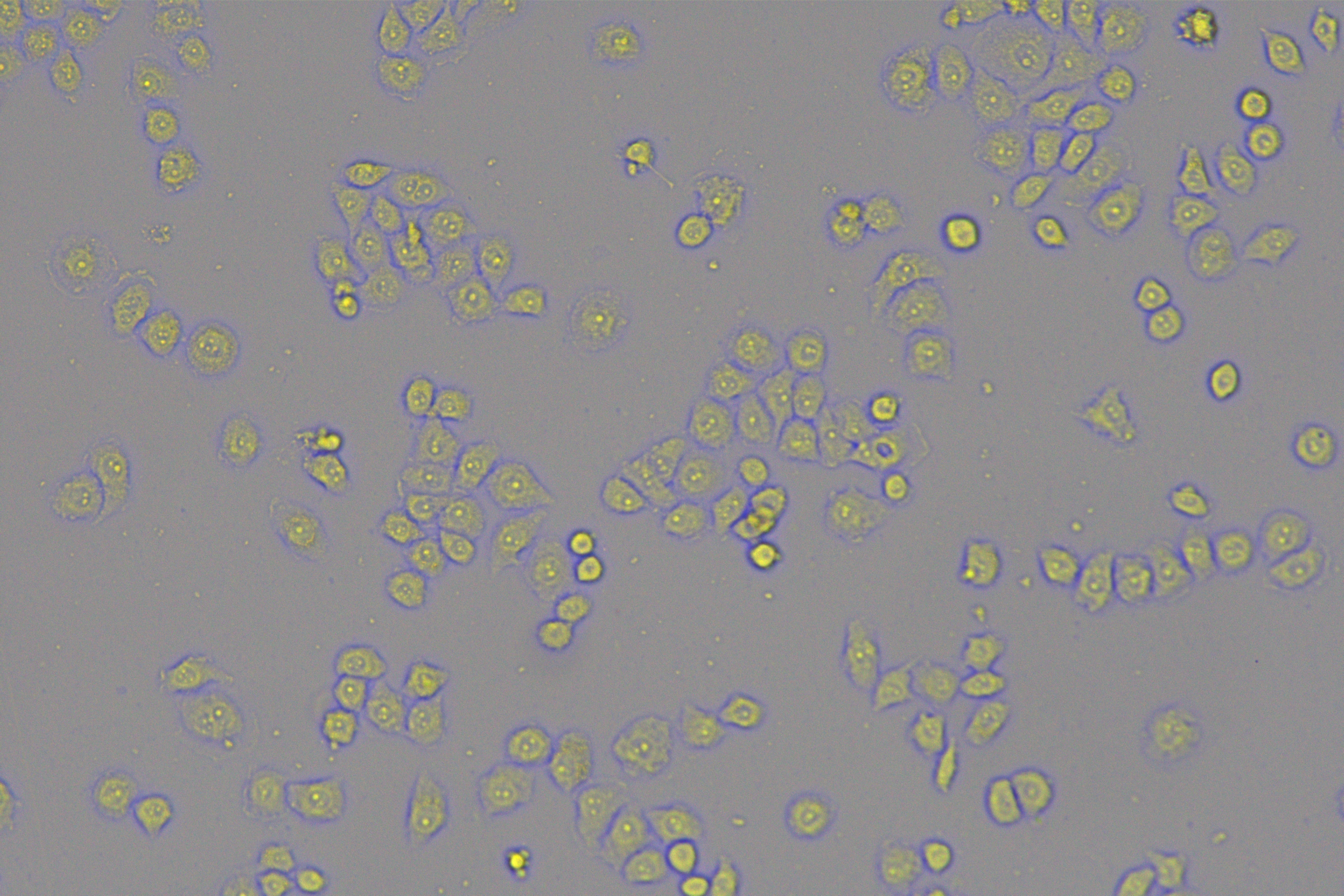

Supplement: Supplemental Information 36 [file peerj-10-12802-s036.jpg]

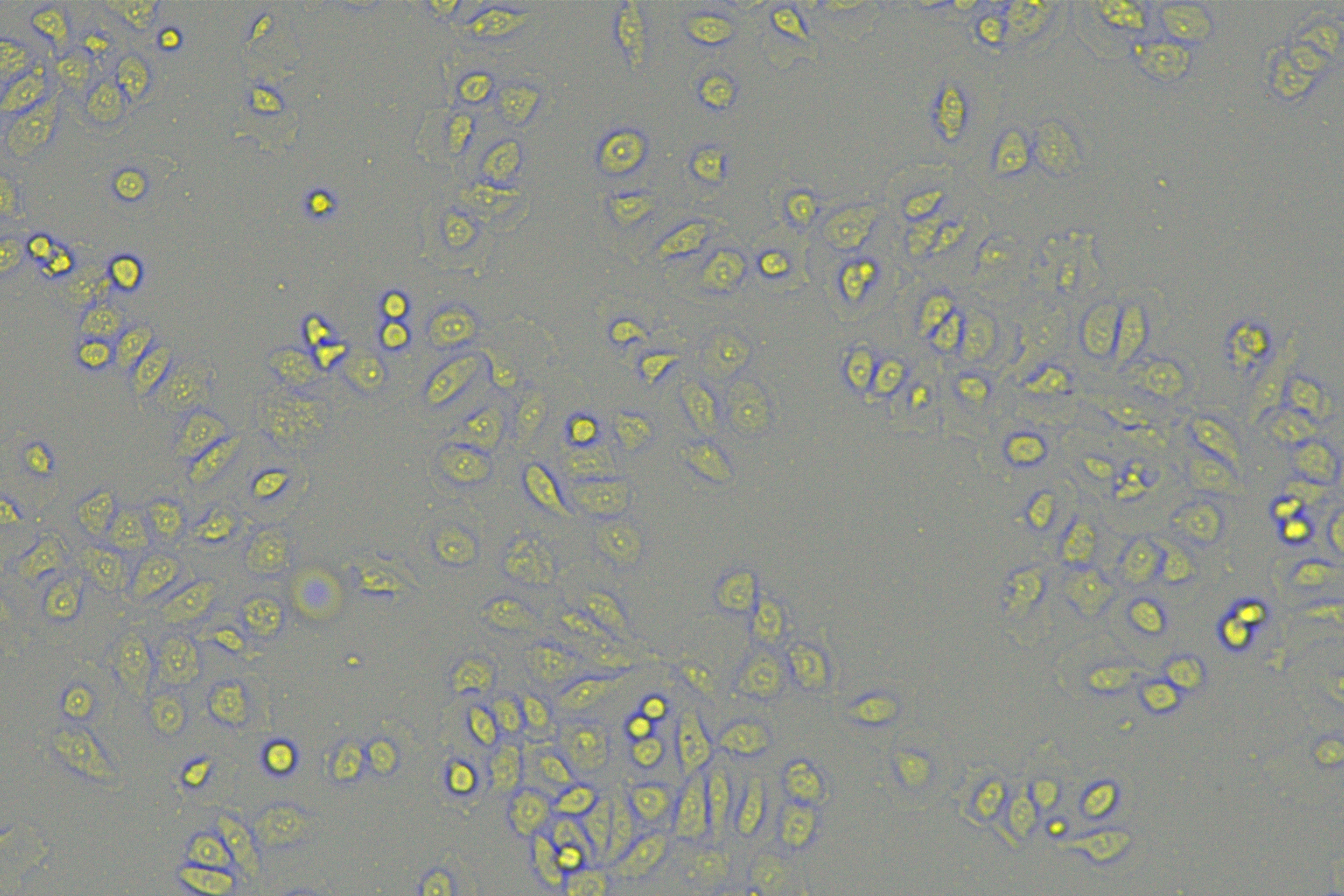

Supplement: Supplemental Information 37 [file peerj-10-12802-s037.jpg]

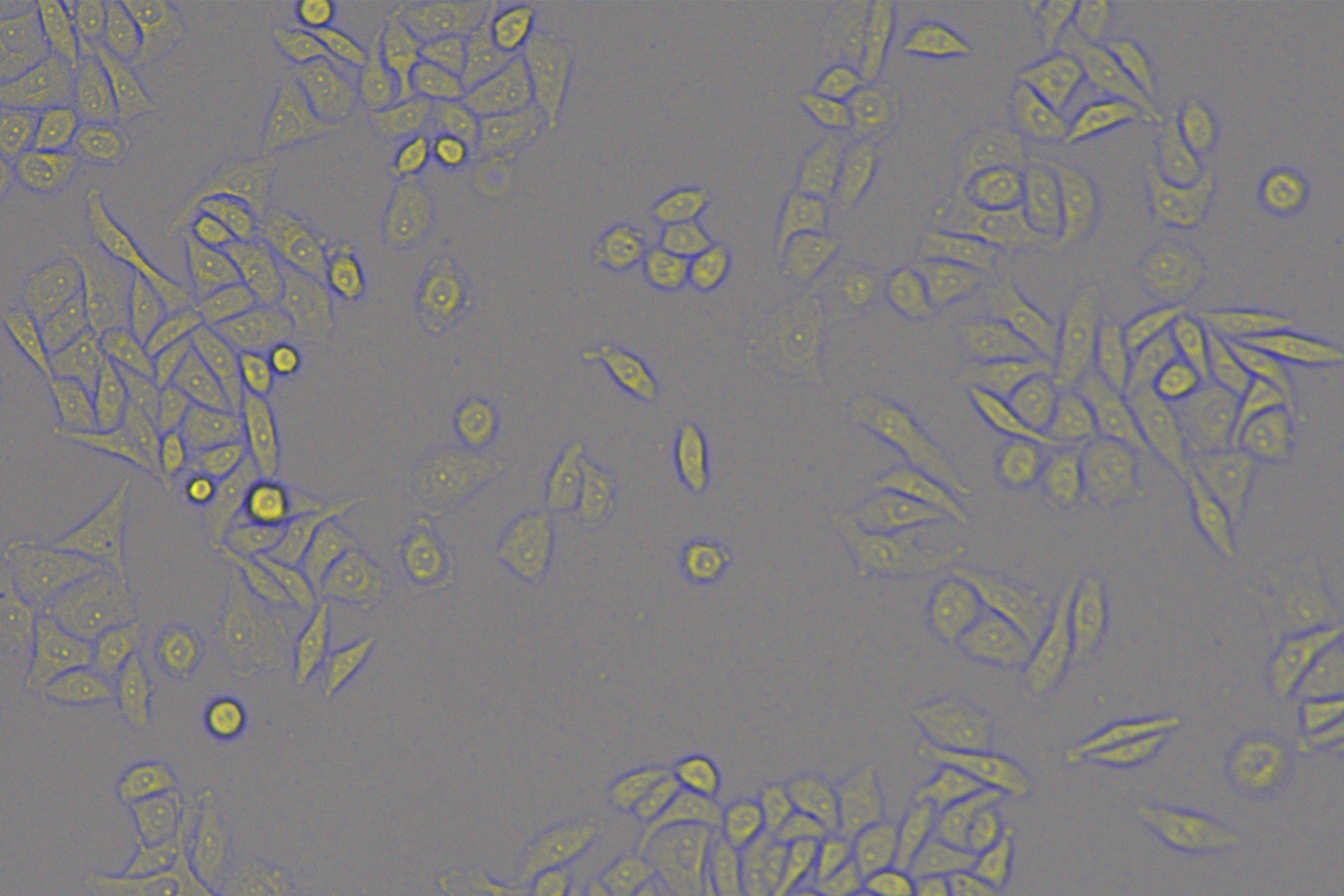

Supplement: Supplemental Information 38 [file peerj-10-12802-s038.jpg]

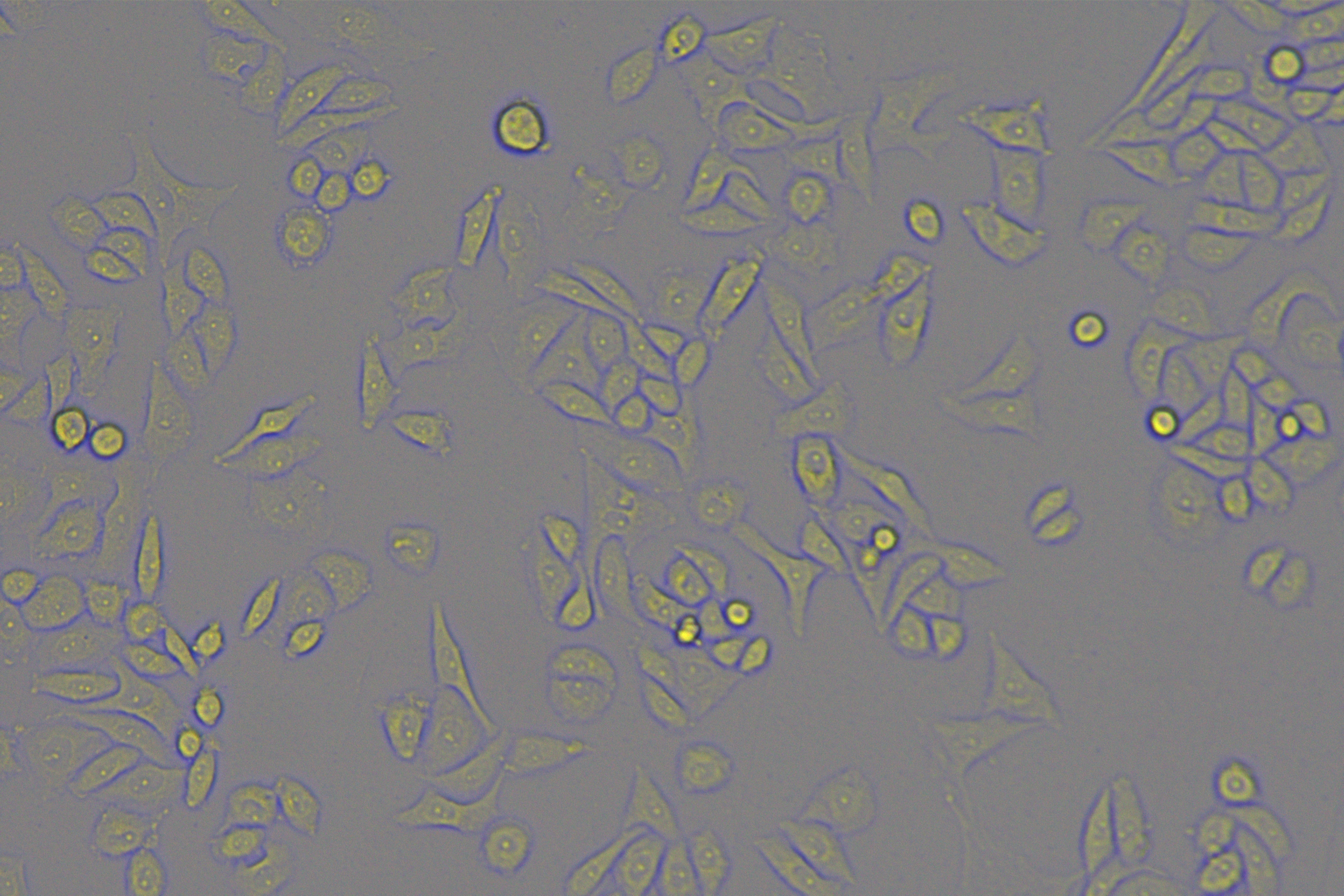

Supplement: Supplemental Information 39 [file peerj-10-12802-s039.jpg]

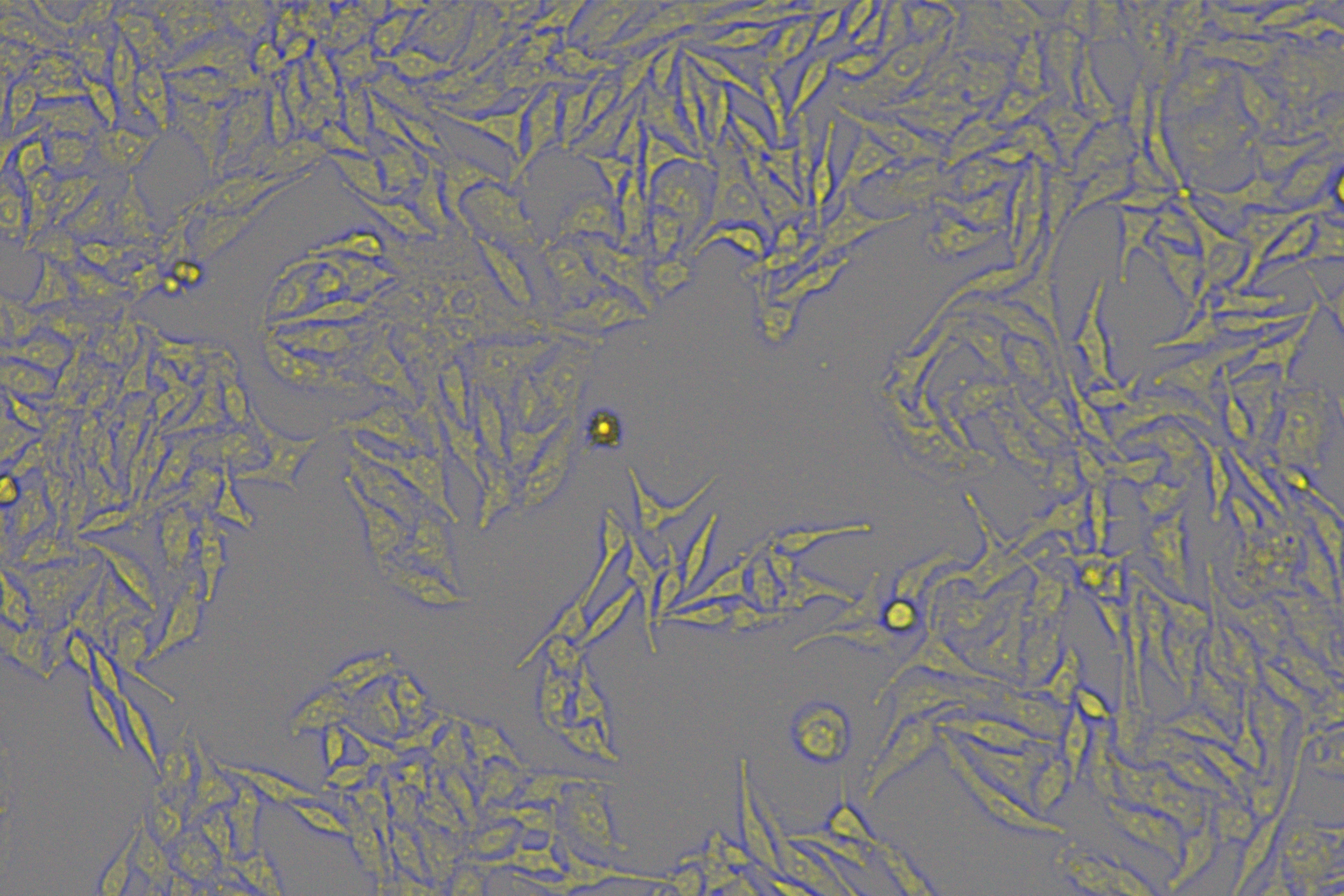

Supplement: Supplemental Information 40 [file peerj-10-12802-s040.jpg]

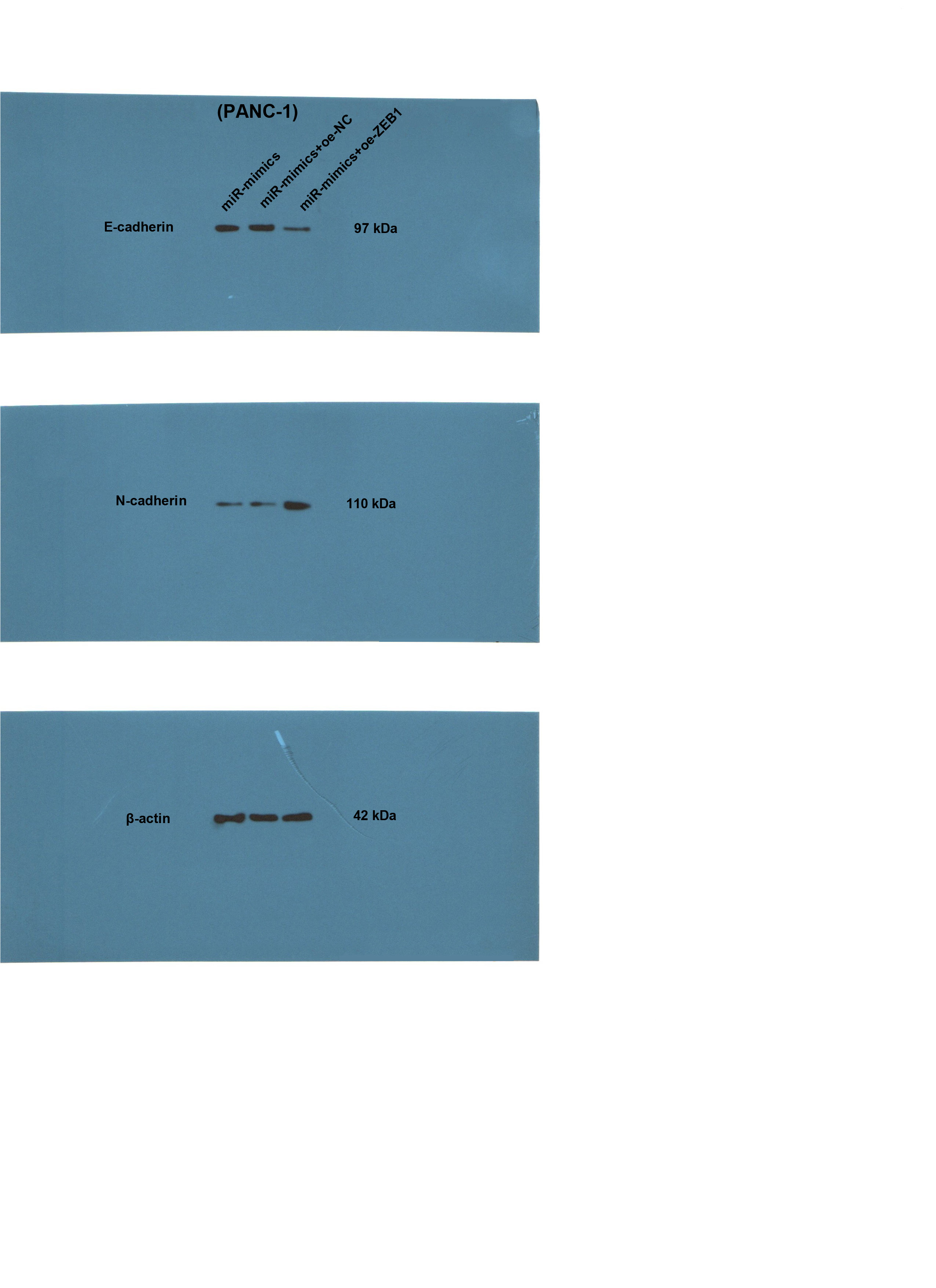

Supplement: Supplemental Information 41 [file peerj-10-12802-s041.jpg]

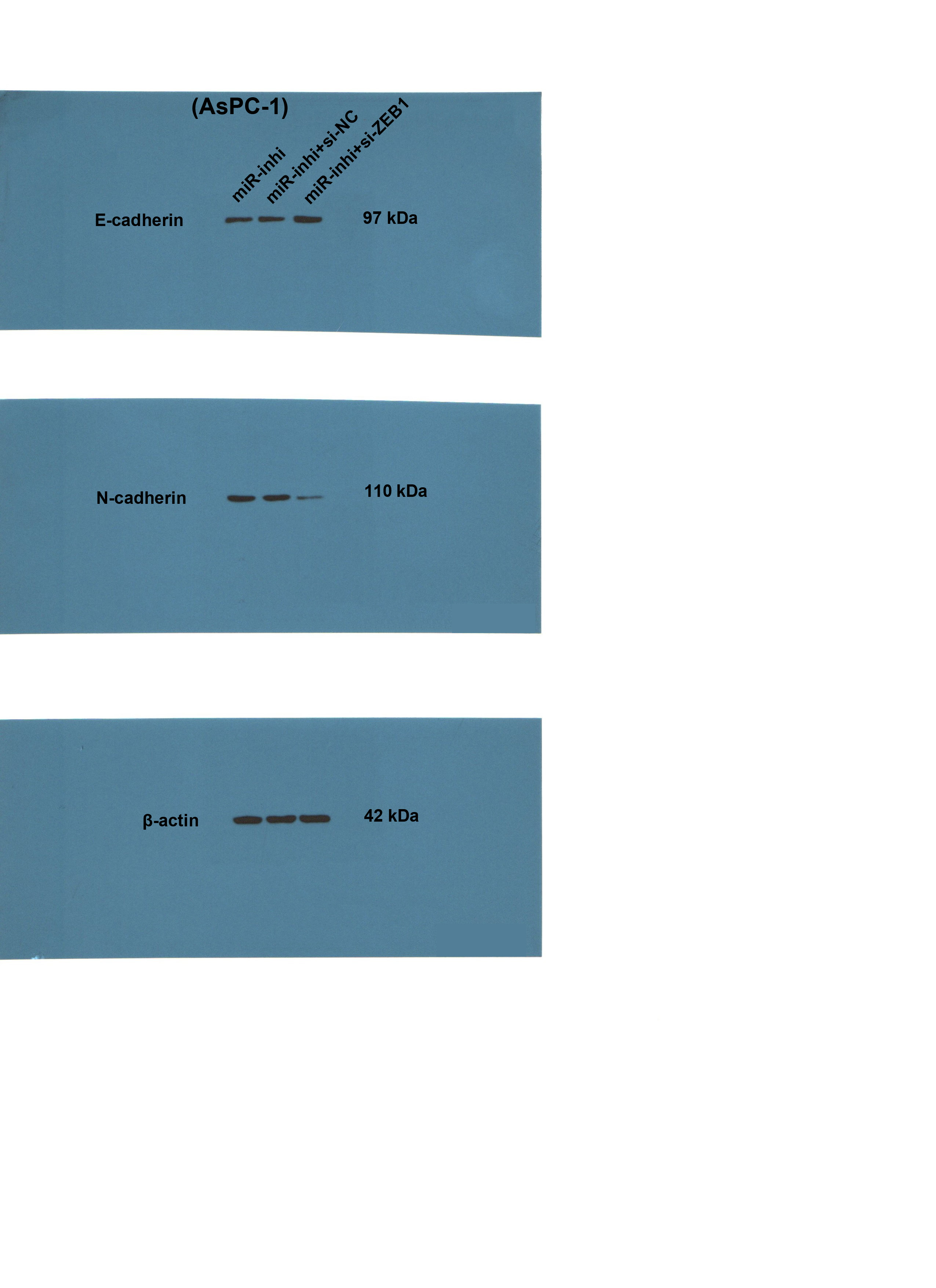

Supplement: Supplemental Information 42 [file peerj-10-12802-s042.jpg]

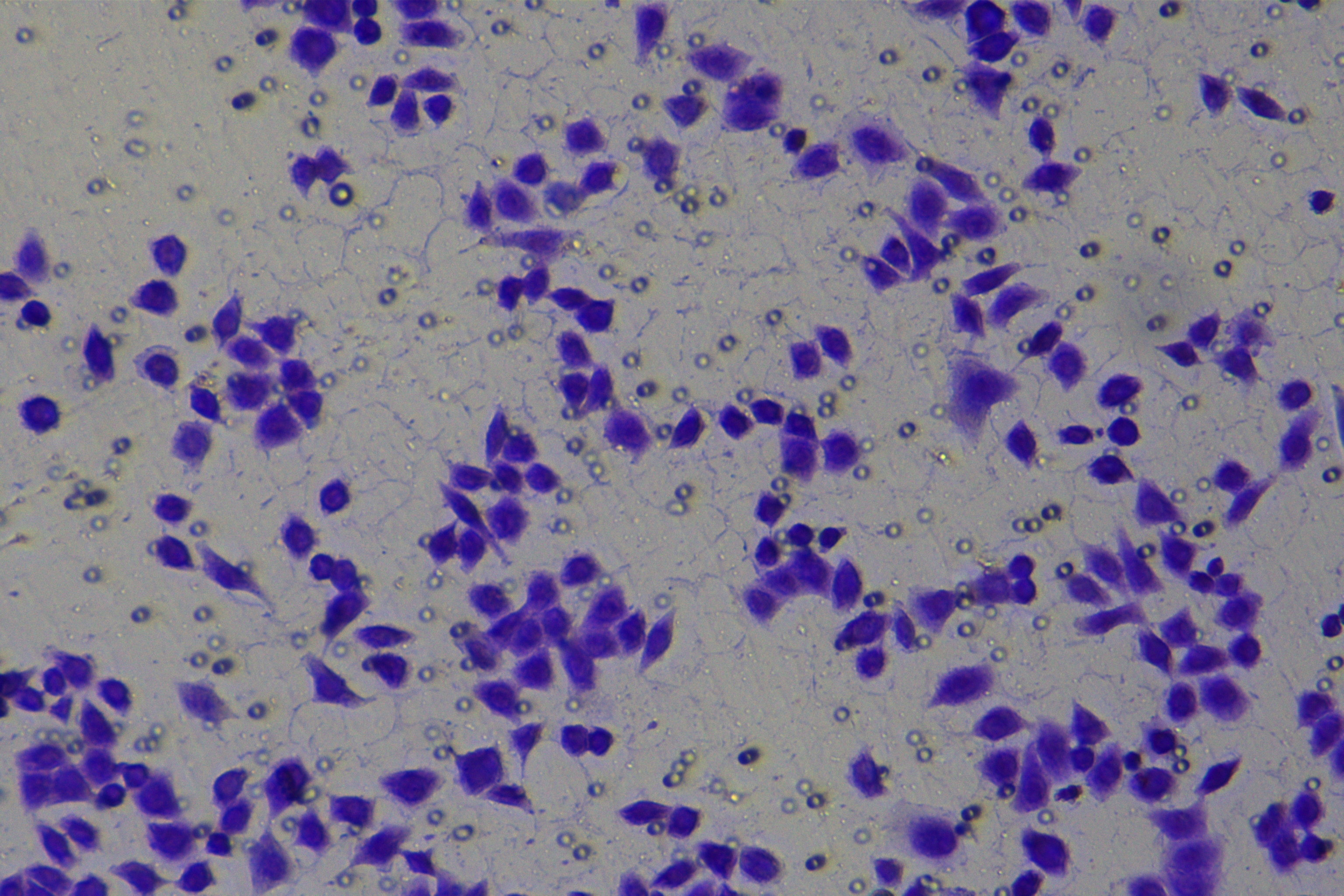

Supplement: Supplemental Information 43 [file peerj-10-12802-s043.jpg]

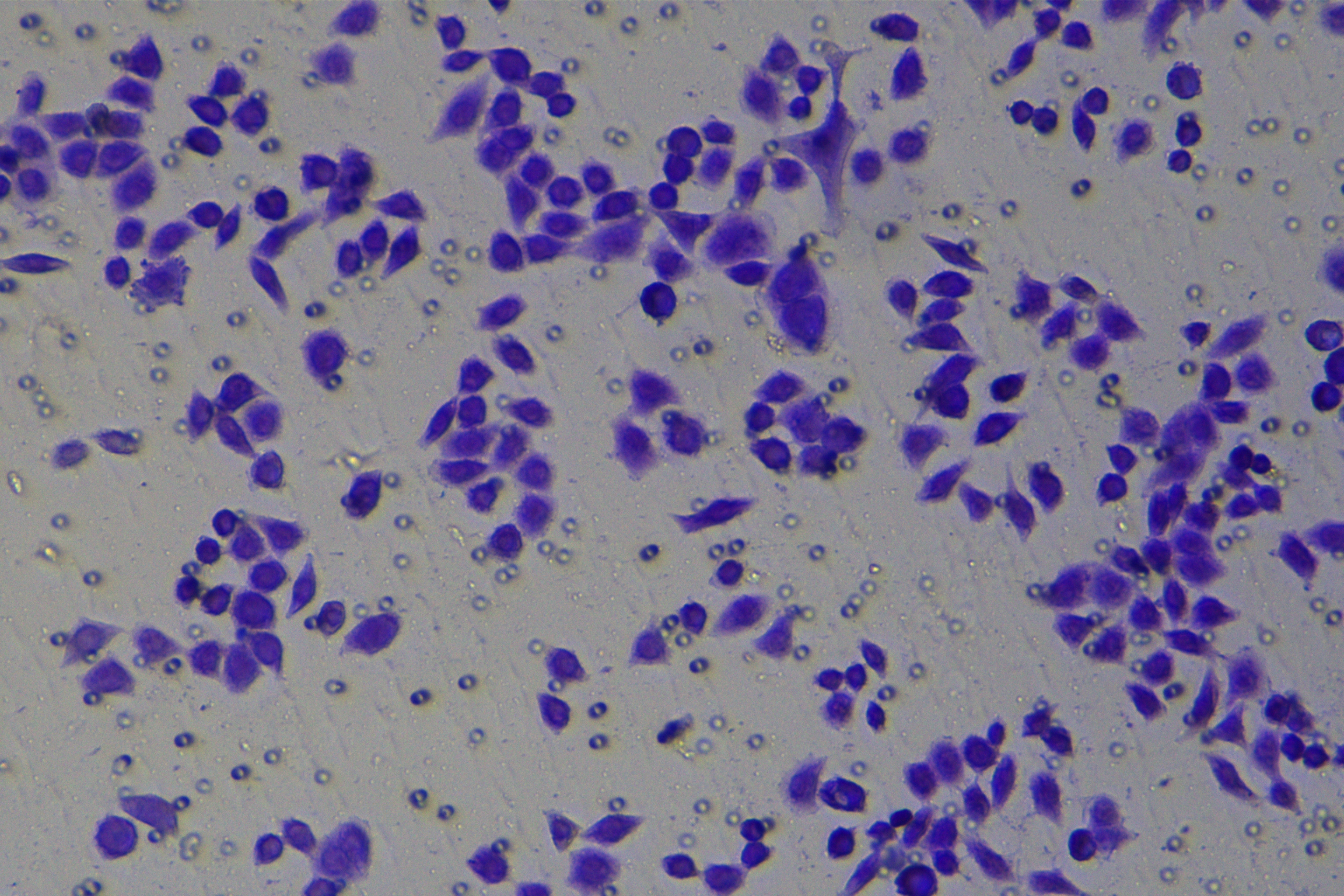

Supplement: Supplemental Information 44 [file peerj-10-12802-s044.jpg]

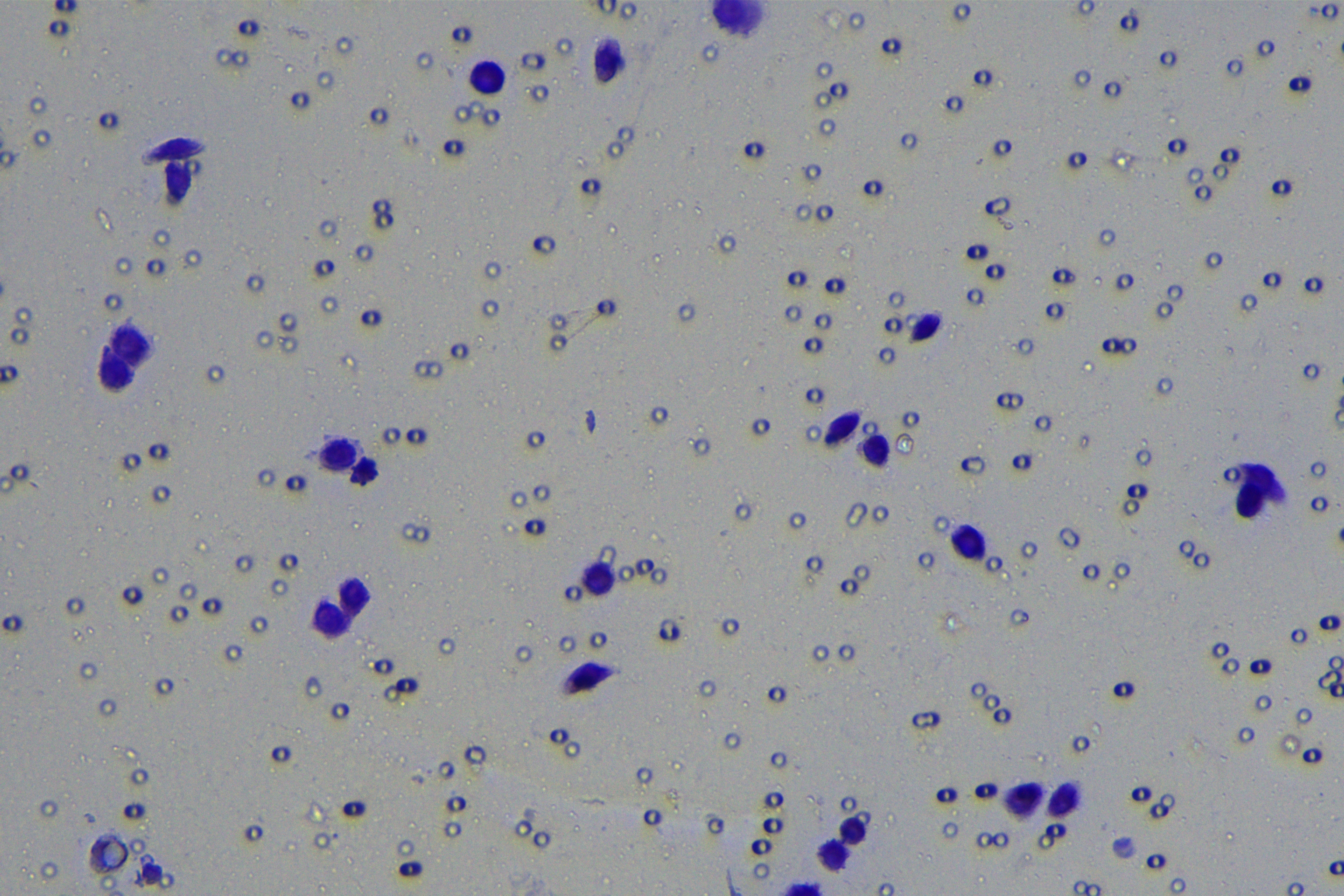

Supplement: Supplemental Information 45 [file peerj-10-12802-s045.jpg]

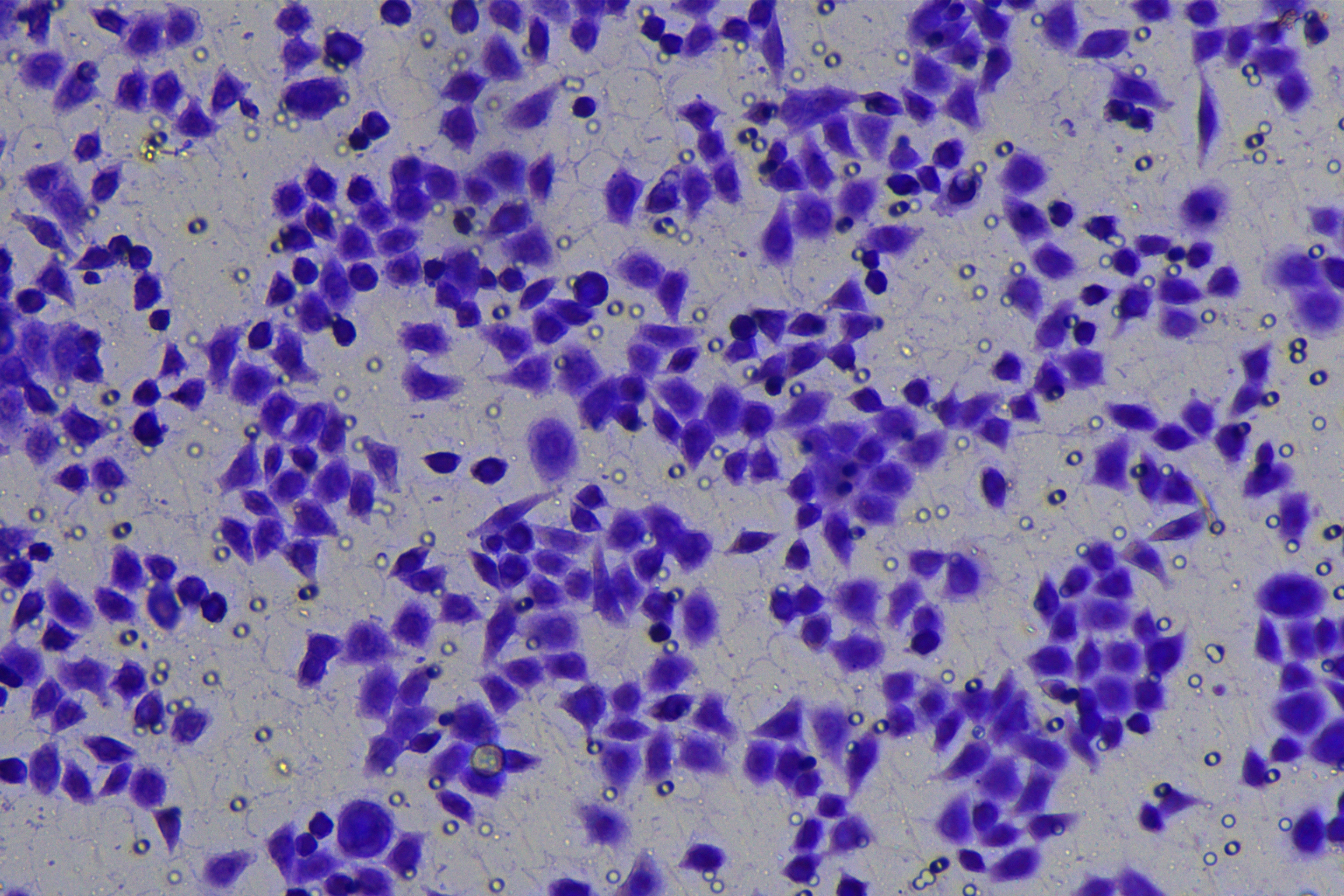

Supplement: Supplemental Information 46 [file peerj-10-12802-s046.jpg]

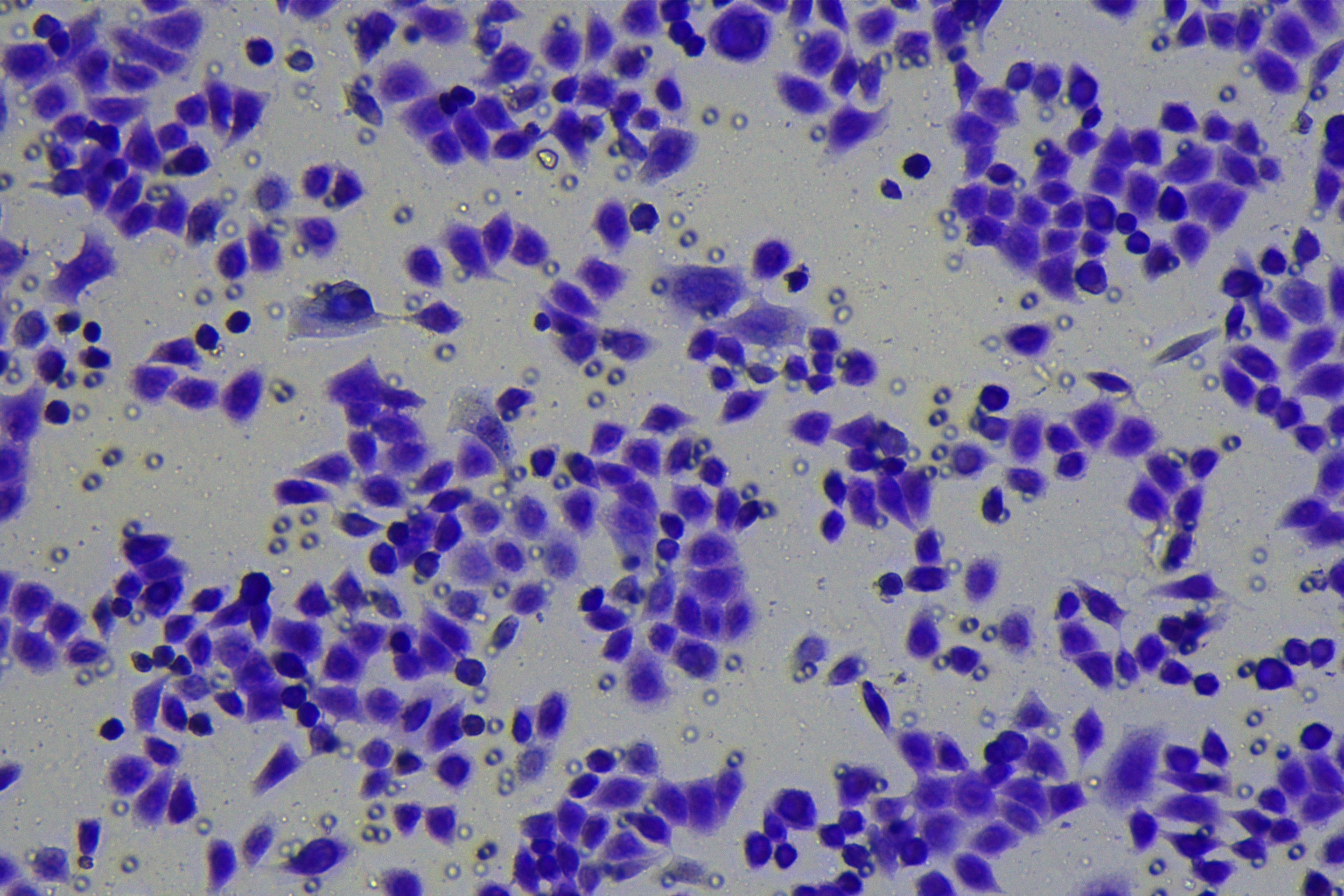

Supplement: Supplemental Information 47 [file peerj-10-12802-s047.jpg]

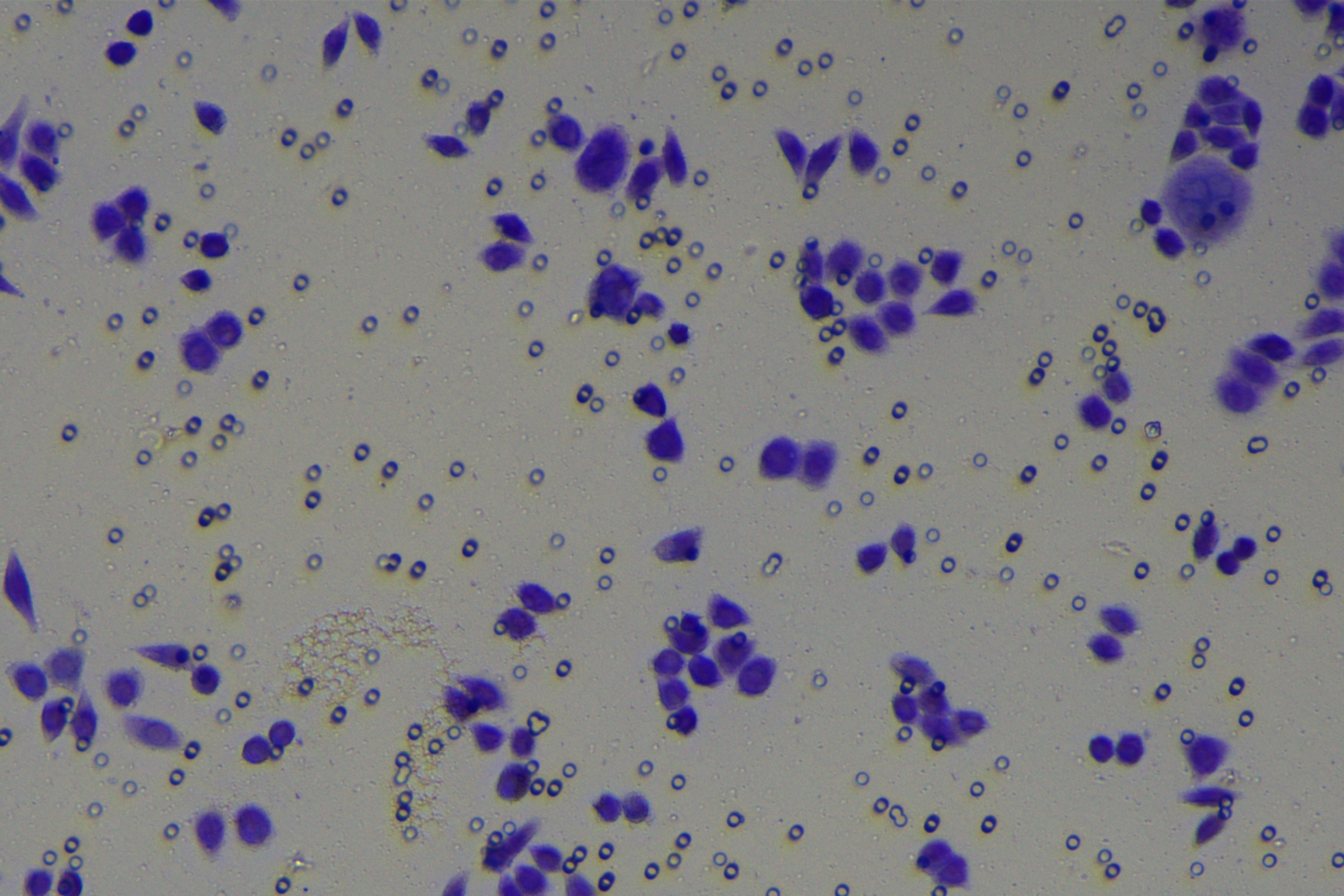

Supplement: Supplemental Information 48 [file peerj-10-12802-s048.jpg]
